# Australindolones, New Aminopyrimidine Substituted Indolone Alkaloids from an Antarctic Tunicate *Synoicum* sp.

**DOI:** 10.3390/md20030196

**Published:** 2022-03-08

**Authors:** Sofia Kokkaliari, Kim Pham, Nargess Shahbazi, Laurent Calcul, Lukasz Wojtas, Nerida G. Wilson, Alexander D. Crawford, Bill J. Baker

**Affiliations:** 1 Department of Chemistry, University of South Florida, 4202 E. Fowler Ave., CHE205, Tampa, FL 33620, USA; skokkaliari@cop.ufl.edu (S.K.); kim.pham@ochsner.org (K.P.); calcul@usf.edu (L.C.); lwojtas@usf.edu (L.W.); 2 Department of Preclinical Sciences and Pathology, Norwegian University of Life Sciences (NMBU), 1430 Ås, Norway; nargess.shahbazi@uibk.ac.at (N.S.); crawford@biodiscoveryinstitute.org (A.D.C.); 3 Research & Collections, Western Australia Museum, 49 Kew Street, Welshpool, WA 6106, Australia; nerida.wilson@museum.wa.gov.au; 4 School of Biological Sciences, University of Western Australia, 35 Stirling Highway, Crawley, WA 6009, Australia; 5 Institute for Arctic and Antarctic Biodiscovery, Medford, OR 97504, USA

**Keywords:** ascidians, indole alkaloids, zebrafish, meridianins

## Abstract

Five new alkaloids have been isolated from the lipophilic extract of the Antarctic tunicate *Synoicum* sp. Deep-sea specimens of *Synoicum* sp. were collected during a 2011 cruise of the R/V *Nathanial B. Palmer* to the southern Scotia Arc, Antarctica. Crude extracts from the invertebrates obtained during the cruise were screened in a zebrafish-based phenotypic assay. The *Synoicum* sp. extract induced embryonic dysmorphology characterized by axis truncation, leading to the isolation of aminopyrimidine substituted indolone (**1**–**4**) and indole (**5**–**12**) alkaloids. While the primary bioactivity tracked with previously reported meridianins A–G (**5**–**11**), further investigation resulted in the isolation and characterization of australindolones A–D (**1**–**4**) and the previously unreported meridianin H (**12**).

## 1. Introduction

Marine invertebrates have been the source of a multitude of bioactive compounds in recent years, with interest drawn especially to sponges and tunicates [1,2,3]. Tunicates can be found in both shallow and deep-water habitats, and due to their extensive diversity, they can potentially be an important resource for biodiscovery [4,5]. Furthermore, only a small number of deep-water tunicates have been analyzed chemically due to the difficulties in accessing deep-sea habitats. Most of the compounds isolated from tunicates are nitrogen-containing, with the most common being aromatic alkaloids and macrocyclic metabolites [6,7,8,9,10].

Species of the genus *Synoicum* have been found in both shallow and deep-water around the world [11]. Most species of *Synoicum* spp. that have been studied chemically are from tropical shallow waters and only a few are from cold water habitats [9,10,12,13]. The existing literature shows that this genus of ascidians produce a variety of secondary metabolites, which are structurally diverse and include, but are not limited to, alkaloids, peptides, and polyketides [14,15,16,17]. These secondary metabolites have shown anti-inflammatory, anti-microbial, and cytotoxic activity [10,14,15,16,17,18].

Most marine secondary metabolites are from shallow tropical and temperate waters due to the ease of access [19,20,21]. In contrast, less than 3% of the reported organisms are from polar habitats, as they were once believed to lack biodiversity [19,22,23]. Antarctica is one of the polar environments that in recent years has been increasingly attracting interest and has been the source of multiple bioactive metabolites. The two major contributors to Antarctica’s biodiversity are the Antarctic Circumpolar Current, which has functioned as a barrier creating biogeographic isolation of the species found there, and periods of glaciation, which have periodically separated benthic populations in refugia, resulting in speciation and/or the generation of different phenotypes within the same species [24,25].

Secondary metabolites from Antarctic invertebrates have seemingly co-evolved with new phenotypes [26], resulting in new and novel scaffolds that have often demonstrated activity in various assays modeling human disease. Zebrafish are an established in vivo platform for disease modeling and drug discovery, and are valuable screening tools for a variety of indication areas including neurological, cardiovascular, metabolic, and infectious diseases and cancer [27,28,29]. Zebrafish are also widely used for toxicity analysis of small molecules, with zebrafish-based assays enabling high-throughput in vivo screening for both drug-induced organ toxicity and developmental toxicity [30,31]. Advantages of zebrafish include their genetic and physiological similarities with humans, and the small size and rapid ex utero development of their embryos and larvae, with which most screens are performed. Over the past decade, zebrafish have also proven their utility for biodiscovery (identification of bioactive natural products), in particular for bioassay-guided isolation from complex extracts [32,33,34,35]. More recently, zebrafish assays have been used for marine biodiscovery [36].

**Figure 1:**
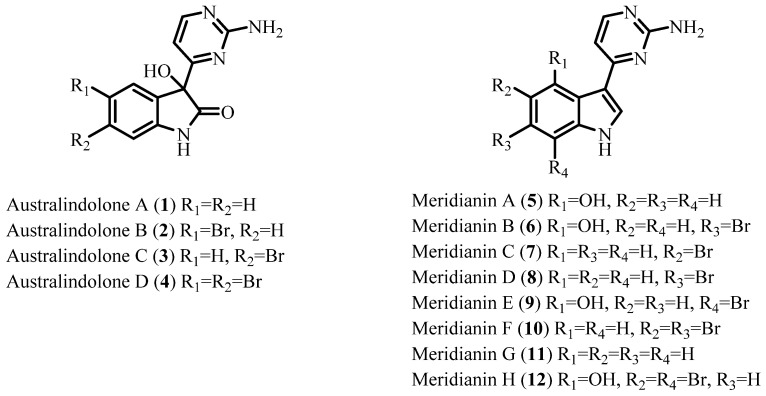
Structures of australindolones A–D (**1**–**4**) and meridianins A–H (**5**–**12**).

In our continuing search for new bioactive compounds from cold-water habitats, an extract of the tunicate *Synoicum* sp. collected in Antarctica was screened in a phenotypic zebrafish assay [37]. This bioassay utilizes the rapid and ex vivo development of zebrafish embryos to enable monitoring of phenotypic changes caused by extracts, fractions, and compounds of interest. Incubation of developing zebrafish embryos with the extract from *Synoicum* sp. induced a distinct embryonic dysmorphology characterized by the truncation of the anterior–posterior axis. *Synoicum* sp. extract-treated embryos exhibited truncation of the trunk and tail and overall developmental delay, indicating the potential involvement of multiple signaling pathways known to be important for embryonic development and anterior–posterior axis specification [37], therefore prioritizing this extract for further investigation. In this paper we report the isolation of four new indolone alkaloids, australindolones A–D (**1**–**4**), as well as the isolation of a new indole alkaloid, meridianin H (**12**), and the previously reported meridianins A–G (**5**–**11**) (Figure 1). The isolation was guided using ^1^H NMR spectroscopy and the zebrafish bioassay. While the bioactivity was tracked with meridianins, the australindolones A–D (**1**–**4**) were obtained as new chemotypes from the tunicate.

## 2. Results and Discussion

*Synoicum* sp. was collected by trawling at a depth of 200 m near Shag Rocks and South Georgia in the Southern Ocean, Antarctica. The sample was extracted and then screened against various biological targets. The non-polar extract was identified as a hit in a developmental zebrafish screening, resulting in the extract to be further investigated. Using bioassay guided fractionation, the active MPLC fractions of the extract were further purified using HPLC, resulting in the isolation of australindolones A–D (**1**–**4**) and meridianins A–H (**5**–**12**) (Figure 1).

### 2.1. Australindolones A–D (***1***–***4***)

Australindolone A (**1**), was isolated as a yellow solid. The HRESIMS supported a molecular formula of C_12_H_10_N_4_O_2_, which was corroborated by proton and carbon NMR data (Table 1) recorded in DMSO-*d*_6_. The high degree of unsaturation (DU = 10), as well as the deshielded ^13^C NMR shifts, were characteristic of a heteroaromatic ring system. A number of functional groups were evident in the NMR data, including a broad singlet at δ_H_ 6.74, exchangeable in D_2_O, and a ^13^C NMR shift at δ_C_ 177.4, suggestive of an ester/amide-type carbonyl. The ^13^C NMR shift at δ_C_ 78.1 (C-3) is characteristic of a carbon bearing oxygen and, since there is only one oxygen unassigned, the likely position of an alcohol group. A deshielded proton at δ_H_ 10.41 (H-1) lacked HSQC correlation, placing it on nitrogen.

**Figure 2:**
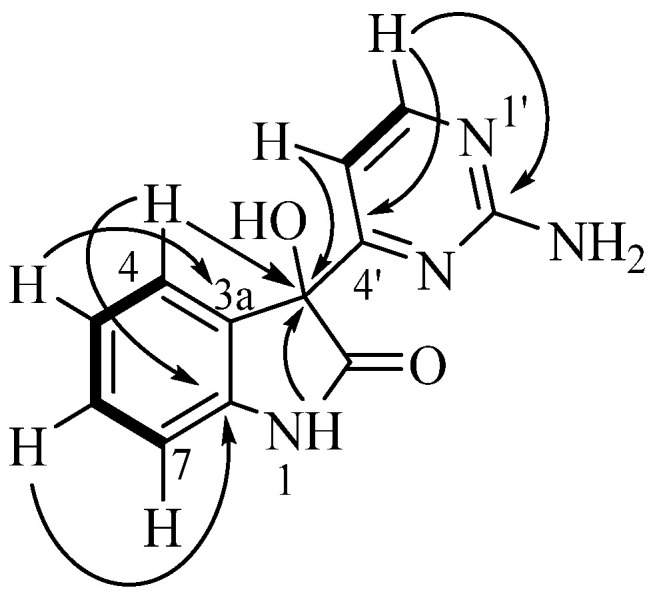
Key COSY (bold) and HMBC (arrows) correlations for indolone **1**.

Analysis of the australindolone A (**1**) 2D NMR data was instructive in developing the scaffold, despite the limited number of protons. The COSY NMR spectrum demonstrated a spin system (Figure 2) establishing the contiguous relationship of δ_H_ 6.99 (H-4), 6.89 (H-5), 7.20 (H-6) and 6.84 (H-7). The HMBC data further extended the scaffold; the combination of H-5 correlating to 133.1 (C-3a), H-4 with δ_C_ 78.1 (C-3), and δ_H_ 10.41 (H-1) to C-3 created a spin system which could be bridged with δ_C_ 177.4 (C-2), creating an indolone skeleton. The shift of H-1 (δ_H_ 10.41), and the HMBC correlation of H-6 to δ_C_ 142.8 (C-7a) supported that assignment. This indolone scaffold has one open valence, at C-3, which was correlated in the HMBC to an additional aromatic system at δ_H_ 6.97 (H-5′) that was COSY correlated to δ_H_ 8.28 (H-6′). Besides the COSY to H-5′, H-6′ demonstrated HMBC correlation to δ_C_ 170.6 (H-4′). This extended indolone skeleton accounted for C_11_H_8_NO_2_ and all COSY correlations; one remaining HMBC relationship was established between H-6′ and the last unaccounted for carbon at δ_H_ 162.9 (H-2′). To complete the structure of australindolone A, N_3_H_2_ and three degrees of unsaturation needed assignment. Chemical shifts of C-2′, C-4′ and C-6′ matched well with the 2-aminopyrimidine ring systems seen in, for example, the meridianins [6], which were also found in this extract. HMBC correlation of H-6′ to C-2′ was supportive of australindolone A as the C-2/C-3 oxidized derivative of meridianin G (**11**).

**Figure 3:**
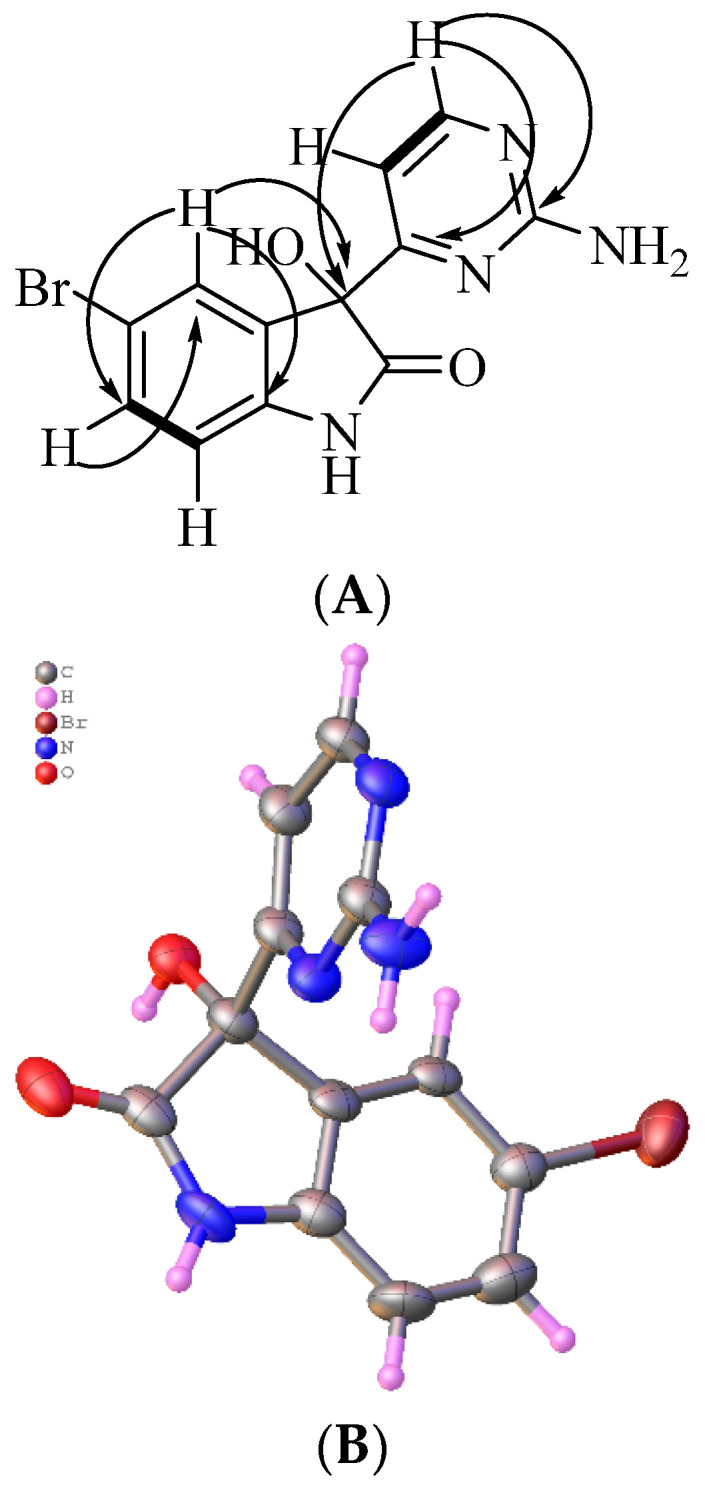
(**A**) Key COSY (bold) and HMBC (arrows) correlations for australindolone B (**2**). (**B**) X-ray structure of **2**. Water and DMSO co-crystallized with australindolone B were removed for clarity; only one out of two enantiomers is shown.

Australindolone B (**2**) was also isolated as a yellow solid. The HRESIMS of **2** established a molecular formula of C_12_H_9_N_4_O_2_Br, supported by the ^1^H and ^13^C NMR data. The NMR shifts supported the existence of a heteroaromatic ring system similar to **1**, but with the presence of a bromine atom. The functional groups present in the molecule were once again established as a carbon bearing oxygen at δ_C_ 78.1 (C-3), an amide carbonyl at δ_c_ 176.9 (C-2), and a proton on nitrogen at δ_H_ 10.57 (H-1). The COSY NMR spectrum showed the vicinal relationship between a proton at δ_H_ 7.39 (H-6) and δ_H_ 6.81 (H-7). The HMBC correlation of H-7 to a carbon at δ_C_ 135.4 (C-3a) and that of H-4 to a carbon at δ_C_ 142.2 (C-7a) and to C-3, extended the scaffold. From the ^1^H NMR data, the presence of two *meta*-oriented protons, δ_H_ 7.12 (H-4), H-6 (*J* = 8.2, 2.1 Hz) and two *ortho*-oriented protons, H-6 and H-7 (*J* = 8.2 Hz) was established. The coupling constants indicated a mono-substituted indolone aromatic ring, with the bromine in either position C-5 (δ_C_ 113.2) or C-6 (δ_C_ 132.0). The correlation from H-6 to C-7a established the bromine in position C-5. Further, the deshielded shift of C-3a (δ_C_ 135.4) combined with the deshielded shift of C-4 (δ_C_ 126.8) and the HMBC correlations of H-6 to C-7a (δ_C_ 142.2), strengthened the positioning of the Br on C-5. The second ring system was created based on the COSY correlations of δ_H_ 6.99 (H-5′) and δ_H_ 8.30 (H-6′). The HMBC correlations of H-6′ to a carbon at δ_c_ 162.9 (C-2′) and one at δ_c_ 170.0 (C-4′) assisted in assigning the 2-aminopyrimidine ring, positioned on C-3 based on the HMBC correlation of H-5′ to C-3 (Figure 3A). Australindolone B provided crystals suitable for X-ray analysis that supported the structure assignment (Figure 3B); the alkaloid crystalizes in the Pbcn centrosymmetric space group as a racemate.

Australindolone C (**3**), a yellow solid similar to other members of this indolone family, displayed a molecular formula of C_12_H_9_N_4_O_2_Br, based on HRESIMS, ^1^H, and ^13^C NMR data. The chemical shift and the 2D NMR data indicated it as isomeric to **2**. The coupling pattern of two *ortho*-oriented protons H-4 (δ_H_ 6.95, d, *J* = 7.8 Hz) and H-5 (δ_H_ 7.08, dd, *J* = 8.1, 2.0 Hz), and two *meta*-oriented protons, H-5 and H-7 (δ_H_ 6.99, d, *J =* 2.0 Hz), indicated once again the presence of the bromine in either position C-5 (δ_C_ 124.2) or C-6 (δ_C_ 121.8). The shielded shift of the 3-OH (δ_H_ 6.85) when compared to **2** and combined with the upfieldshielded shift of H-4 (δ_H_ 6.95) indicated that the position of the Br is on C-6. Further confirmation is given by the deshielded shift of C-7a (δ_C_ 144.6) and shielded shift of C-3a (δ_C_ 132.5), as well as the shielded shift of C-6 when compared to **2** and the HMBC correlation of H-5 to C-3a.

**Figure 4:**
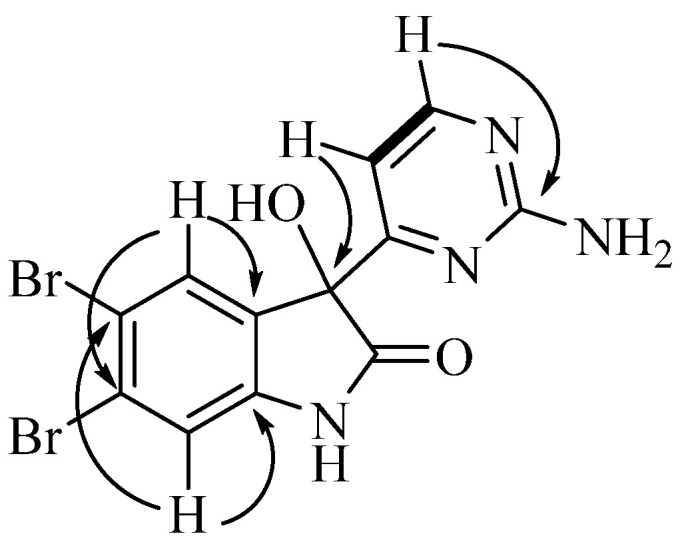
Key COSY (bold) and HMBC correlations for australindolone D (**4**).

Australindolone D (**4**) was isolated as a yellow solid and the molecular formula was determined as being C_12_H_8_N_4_O_2_Br_2,_ based on HRESIMS and supported by the 1D NMR data. The lack of COSY correlations combined with the presence of exchangeable protons with no HSQC correlation complicated the structure elucidation. The HMBC correlations of the protons at δ_H_ 7.32 (H-4) and at δ_H_ 7.21 (H-7) to the carbons at δ_C_ 115.8 (C-5), δ_C_ 124.6 (C-6), and δ_C_ 144.0 (C-7a) indicated the existence of an aromatic ring. The HMBC correlation of H-4 to a carbon at δ_c_ 135.2 (C-3a) assisted in closing the ring. The multiplicity of H-4 (s) and H-7 (s) suggested the positioning of the bromines being in positions 4 and 6, 5 and 6, or 5 and 7. The HMBC correlation of H-4 to an oxygen bearing carbon at δ_C_ 78.3 (C-3) indicated that the two bromines could not be in positions 4 and 7. Using the shielded shift of C-5 and C-6, as well as the deshielded shift of H-4, H-7 and the proton at δ_H_ 7.00 (3-OH) the two bromine atoms were placed in positions C-5 and C-6 and the ring was bridged with the amide type bond between the proton at δ_H_ 10.74 (1-NH), supported by a carbon resonance at δ_C_ 177.2 (C-2). Next the correlation between a proton at δ_H_ 7.00 (H-5′) and one at δ_H_ 8.33 (H-6′) was the only COSY correlation observed. H-5′ and H-6′ showed HMBC correlations to a carbon at δ_C_ 169.9 (C-4′), while H-6′ also showed a correlation to a carbon at δ_C_ 163.4 (C-2′), creating the 2-aminopyrimidine ring similar to the other australindolones (**1**–**3**). The HMBC correlation of H-5′ and H-6′ to C-3 connected the two partial structures (Figure 4).

### 2.2. Meridianins A–H (***5***–***12***)

Meridianins A–G (**5**–**11**), which were first isolated from the ascidian *Aplidium meridianum*, were isolated as yellow solids, with meridianin E (**9**) being the major secondary metabolite of the extract [6,7,8,9,10]. The molecular formula of the compounds were established using HRESIMS as C_12_H_10_N_4_O for **5,** C_12_H_9_N_4_OBr for **6** and **9,** C_12_H_9_N_4_Br for **7** and **8**, C_12_H_8_N_4_Br_2_ for **10**, and C_12_H_10_N_4_ for **11**. Comparison of the ^1^H NMR data (Figures S25–S40) to the literature values assisted in assigning the structures as meridianins A–G (**5**–**11**) [6,7,8,9,10].

**Figure 5:**
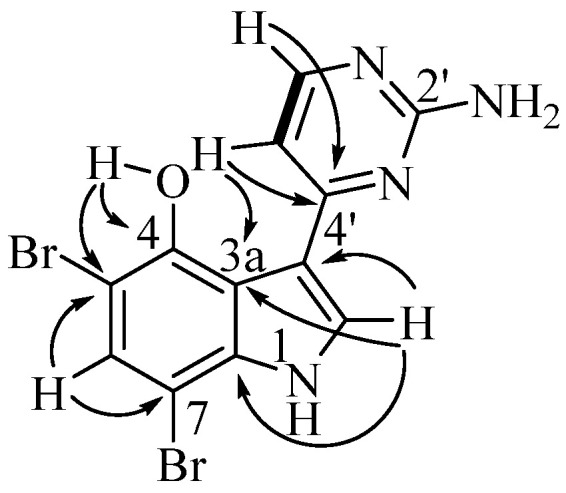
Key COSY (bold) and HMBC correlations for meridianin H (**12**).

Meridianin H (**12**) was isolated as a yellow solid, with the HRESIMS indicating a molecular formula of C_12_H_8_N_4_OBr_2._ The ^1^H and ^13^C NMR spectra in DMSO-*d*_6_ indicated the presence of heteroaromatic shifts (Table 2). HMBC correlations of δ_H_ 7.23 (H-5′) and δ_H_ 8.17 (H-6′) to δ_C_ 159.3 (C-4′), H-6′ to δ_C_ 104.6 (C-5′), H-5′ to δ_C_ 159.4 (C-6′), and H-5′/H-6′ COSY correlation supported the presence of the heteroaromatic 2′-aminopyrimidine system observed in other meridianins. This was further supported by a broad singlet at δ_H_ 6.91 (2H), characteristic of the amine function. H-5′ further correlated in the HMBC spectrum to δ_C_ 114.7 (C-3), placing the 2-aminopyrimidine. The sharp deshielded signal at δ_H_ 15.07 (s), which is absent in the CD_3_OD spectrum, indicated the presence of a phenol, which displayed an HMBC correlation to δ_C_ 116.3 (C-3a), δ_C_ 148.7 (C-4), and δ_C_ 99.1 (C-5). Further HMBC correlations were observed between δ_H_ 8.34 (H-2), δ_C_ 114.7 (C-3), C-3a, and δ_C_ 136.1 (C-7a), which, similarly to the 2-aminopyrimidine ring system, correlates well with other meridianin pyrrole/indole rings. The indole ring system can be completed by observation of HMBC correlations from δ_H_ 7.42 (H-6) to quaternary aromatic carbons C-4, C-5, and δ_C_ 93.0 (C-7). The two bromine atoms in the molecular formula fill the last two open valences (Figure 5).

### 2.3. On the Stereochemistry of the Australindolones

All of the australindolones (**1**–**4**) produced very small but consistent optical rotations (−7 to −12 degrees). This contrasts with the crystal analyzed by XRD, which was racemic. Complicating the discussion, at the concentration tested, these rotations are near the limit of detection of the polarimeter. However, small rotations, including several with rotations under 10 degrees (absolute value) have been reported for 3-substituted oxindolones [37]. Whether the australindolones are racemic or scalemic remains to be determined.

### 2.4. Bioactivity of the Aminopyrimidines

**Figure 6:**
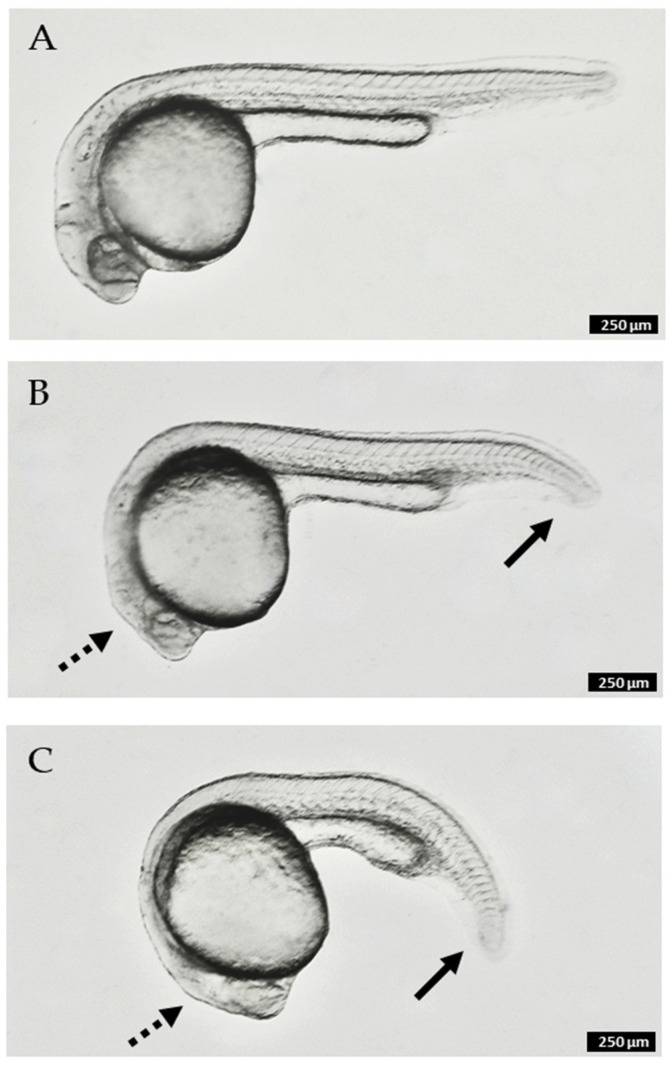
Zebrafish embryos at 24 h post-fertilization (hpf) after 20 h of compound treatment: (**A**). control (no treatment); (**B**). 300 µM australindolone D (**4**); (**C**). 60 µM meridianin H (**12**). Solid arrows indicate partial truncation of posterior structures (tail); dashed arrows indicate reduction in size of anterior structures (head). Scale bar 250 µm.

Our investigation of the chemistry of *Synoicum* sp. was initiated based on activity of the crude extract using a zebrafish developmental model. Purified meridianins (**5**–**11**) were found with the most potent effect, in which embryos showed truncation of the anterior–posterior axis (e.g., Figure 6C), which was observed by the curling of the tail and body when compared to the negative control (Figure 6A), as well as the lack of proper elongation of the tail and the incomplete growth of the main body [38]. Other observations made include necrosis, observed as darkened spots under the microscope at different parts of the embryo. Australindolones (**1**–**4**) displayed considerably less activity (e.g., Figure 6B). Additional work is currently underway to establish the underlying cause of the observed phenotype in the meridianins.

## 3. Materials and Methods

### 3.1. General Experimental Procedures

A Rudolph Research (Hackettstown, NJ, USA) Autopol IV polarimeter was used to measure the optical rotation at 589 nm. IR spectra were measured using an Agilent Technologies (Santa Clara, CA, USA) Cary 630 FTIR. UV spectra were measured using an Agilent Technologies (Santa Clara, CA, USA) Cary 60 UV-Vis spectrophotometer. A Varian Innova 500, Varian Direct Drive 500, or Varian Innova 400 MHz NMR spectrometer (Agilent, Santa Clara, CA, USA) at 298 K was used to record the NMR spectra. The NMR spectra were recorded using as reference the residual non-deuterated shifts from DMSO-*d*_6_ (*δ*_H_ 2.50 ppm and *δ*_C_ 39.51 ppm) (Cambridge Isotopes Laboratory, Tewksbury, MA, USA). The high-resolution mass spectra were recorded on an Agilent Technologies (Santa Clara, CA, USA) LC/MS ToF electrospray ionization spectrometer. MPLC was carried as direct injections on a RediSep C18 50 g flash column using a Teledyne Isco (Lincoln, NE, USA) Combiflash Rf200i, equipped with an evaporative light scattering detector. HPLC was performed using a preparative YMC-Pack (Devens, MA, USA) ODS RP column (250 × 20 mm, 10 µm) and analytical C-18 columns (250 × 10 mm, 5 µm) on a LC-20AD Shimadzu (Columbia, MD, USA) system and an SPD-20A UV detector.

### 3.2. Animal Material

The yellow tunicate *Synoicum* sp. was collected by trawling at a depth of 200 m near Shag Rocks and South Georgia in Antarctica (−42.0188 S, −53.4215 W) and stored at −20 °C until it was analyzed. The organism was identified by Dr. Linda Cole of the Smithsonian Institution (National Museum of Natural History accession number 2059503, http://n2t.net/ark:/65665/305f419e7-84a0-41d7-902e-b7758b253e87 (accessed on 3 February 2022)).

### 3.3. Extraction and Isolation

Frozen *Synoicum* sp. was lyophilized, and 200 g of dry organism were extracted using 1:1 CH_2_Cl_2_/MeOH three times for 24 h each. The extract was dried on a rotary evaporator, and the residue was partitioned between hexane and 95% aqueous MeOH to remove non-polar components. The aqueous layer was concentrated and further partitioned between EtOAc and H_2_O to remove salts. The EtOAc layer was dried, and the 2 g of crude extract were subjected to medium pressure liquid chromatography with a H_2_O/MeOH gradient, collected in 7 fractions. Further purification was performed on HPLC using 5–100% H_2_O/MeCN and a C-18 analytical column, to afford australindolones A (**1**) (2.0 mg), B (**2**) (4.0 mg), C (**3**) (1.0 mg), D (**4**) (2.0 mg), meridianin H (**12**) (2.0 mg), and the known meridianins A–G (**5**–**11**) (A: 2.0 mg, B: 4.0 mg, C: 2.0 mg, D: 3.5 mg, E: 5.0 mg, F: 1.5 mg, G: 1.0 mg); meridianins A–G were identified by comparison with published NMR data. Overall yields of alkaloids were found as 0.001% for **5**, **7**, **12**, **1,** and **4**, 0.002% for **6** and **2**, 0.00175% for **8**, 0.0025% for **9**, 0.00075 for **10**, and 0.0005% for **11** and **3**. All of the alkaloids were isolated as yellow, solids.

Australindolone A (**1**): [α]_D_^21^ = −11 (*c* = 0.1, MeOH); UV (MeOH) *λ*_max_ (log ε): 213 (3.87), 297 (3.19) nm; IR (thin film): 3379, 2929, 1726, 1625, 1577 cm^−1^; ^1^H and ^13^C NMR data, see Table 1; HRESIMS *m*/*z* 243.0866 [M + H]^+^ (calculated 243.0877 for C_12_H_11_N_4_O_2_).

Australindolone B (**2**): [α]_D_^21^ = −7 (*c* = 0.1, MeOH); UV (MeOH) *λ*_max_ (log ε) 211 (3.03), 297 (2.42) nm; IR (thin film): 3361, 1636, 1581 cm^−1^; ^1^H and ^13^C NMR data, see Table 1; HRESIMS *m*/*z* 320.9951 [M + H]^+^ (calculated 320.9982 for C_12_H_10_N_4_O_2_Br).

Australindolone C (**3**): [α]_D_^21^ = −12 (*c* = 0.1, MeOH); UV (MeOH) *λ*_max_ (log ε) 218 (3.99), 303 (3.13) nm; IR (thin film): 3371, 2925, 1737, 1618, 1569 cm^−1^; ^1^H and ^13^C NMR data, see Table 1; HRESIMS *m*/*z* 320.9944 [M + H]^+^ (calculated 320.9982 for C_12_H_10_N_4_O_2_Br).

Australindolone D (**4**): [α]_D_^21^ = −13 (*c* = 0.1, MeOH); UV (MeOH) *λ*_max_ (log ε) 223 (3.79), 298 (3.06) nm; IR (thin film): 3353,1733, 1618, 1584 cm^−1^; ^1^H and ^13^C NMR data, see Table 1; HRESIMS *m*/*z* 398.9059 [M + H]^+^ (calculated 398.9087 for C_12_H_9_N_4_O_2_Br_2_).

Meridianin H (**12**): UV (MeOH) *λ*_max_ (log ε) 223 (3.53), 345 (3.01) nm; IR (thin film): 3402, 2925, 1737, 1625, 1584 cm^−1^; ^1^H and ^13^C NMR data, see Table 2; HRESIMS *m*/*z* 382.9138 [M + H]^+^ (calculated 382.9138 for C_12_H_9_N_4_OBr_2_).

### 3.4. X-ray Diffraction of Australindolone B (***2***)

X-ray diffraction data for australindolone B (**2**) were measured on a Bruker D8 Venture PHOTON 100 CMOS diffractometer equipped with a Cu Kα INCOATEC ImuS micro-focus source (λ = 1.54178 Å). Indexing was performed using APEX4 (Bruker, Madison, WI, USA; Difference Vectors method). Data integration and reduction were performed using SaintPlus (Bruker, Madison, WI, USA). Absorption correction was performed by the multi-scan method implemented in SADABS [39]. Space group was determined using XPREP implemented in APEX3 (Bruker, Madison, WI, USA). Structure was solved using SHELXT [40] and refined using SHELXL-2018/3 [41] (full-matrix least-squares on F2) through the OLEX2 interface program [42]. An ellipsoid plot was drawn with Platon [43]. Minor parts of disorder were refined with restraints. There are several violations of systematic absences in the data that could be due to presence of minor twinning. Refinement of the model solved in lower symmetry space groups did not result in elimination of residual peaks and significant improvement of R-factors. No obvious signs of twinning were detected, residual peaks were modeled as minor disordered part of main molecule. Data and refinement conditions are shown in Table S1. CCDC Deposition Number 2151169.

### 3.5. Bioassay Procedure

Wildtype *Danio rerio* fish were used for the assay. Once the zebrafish eggs were collected, they were placed in fresh media along with methylene blue, to deter fungal growth. The embryos were sorted and placed in a 96-well plate and the volume was standardized. For the purpose of this screening, it was determined that the optimal point to add the compounds was 4 h post-fertilization (hpf) and the ending point of the assay was 72 hpf. The delay in growth and the dysmorphologies were monitored and assessed. The maximum tolerated concentration was identified for each extract and compound. The plates were incubated at 28 °C and examined under a microscope periodically for up to 72 hpf.

## Figures and Tables

**Table 1:** NMR spectroscopic data for compounds **1**–**4** in DMSO-*d*_6_.

	1			2			3			4		
Position	δ_C_^a^	δ_H_^b^	HMBC	δ_C_^a^	δ_H_^b^	HMBC	δ_C_^a^	δ_H_^b^	HMBC	δ_C_^a^	δ_H_^b^	HMBC
1 NH		10.41 (1H, s)	3		10.57 (1H, s)			10.57 (1H, s)			10.74 (1H, s)	
2	177.4, C			176.9, C			177.3, C			177.2, C		
3	78.1, C			78.1, C			77.8, C			78.3, C		
3-OH		6.74 (1H, brs)			6.91 (1H, brs)			6.85 (1H, brs)			7.00 (1H, s)	
3a	133.1, C			135.4, C			132.5, C			135.2, C		
4	124.1, CH	6.99 (1H, d, 7.2)	3, 6, 7a	126.8, CH	7.12 (1H, d, 2.0)	3, 5, 6, 7, 7a	126.0, CH	6.95 (1H, d, 7.8)	3, 6, 7a	129.2, CH	7.32 (1H, s)	3, 3a, 5, 6, 7a
5	121.6, CH	6.89 (1H, dd, 7.5, 7.5)	3a, 7	113.2, C			124.2, CH	7.08 (1H, dd, 8.1, 2.0)	3a, 6, 7	115.8 C		
6	129.4, CH	7.20 (1H, dd, 7.5, 7.5)	4, 7a	132.0, CH	7.39 (1H, dd, 8.2, 2.1)	4, 5, 7a	121.8, C			124.6 C		
7	109.8, CH	6.84 (1H, d, 7.7)	3a, 5	111.9, CH	6.81(1H, d, 8.2)	3, 3a, 4, 5, 7a	112.6, CH	6.99 (1H, d, 2.0)	5, 6, 7a	115.1, CH	7.21 (1H, s)	3a, 5, 6, 7a
7a	142.8, C			142.2, C			144.6, C			144.0, C		
1′				-	-	-	-	-	-	-	-	-
2′	162.9, C			162.9, C			162.9, C			163.4, C		
2′-NH_2_		6.48 (2H, brs)			6.53 (2H, brs)			6.51 (2H, brs)			6.55 (2H, brs)	2′, 4′, 6′
3′				-	-	-	-	-	-	-	-	-
4′	170.6, C			170.0, C			170.1, C			169.9, C		
5′	105.8, CH	6.97 (1H, d, 5.1)	3, 6′	105.8, C	6.99 (1H, d, 5.1)	3, 4′, 6′	105.7, CH	6.97 (1H, d, 4.9)	6′	106.3, CH	7.00 (1H, d, 5.0)	3, 4′, 6′
6′	158.9, CH	8.28 (1H, d, 5.1)	2′, 4′, 5′	159.1, C	8.30 (1H, d, 5.1)	3, 2′, 4′, 5′	159.0, CH	8.30 (1H, d, 4.9)	4′,5′	159.7, CH	8.33 (1H, d, 5.0)	3, 2′, 4′, 5′

^a^ 125 MHz, multiplicity from HSQC; ^b^ 500 MHz (integration, multiplicity, *J* (Hz)).

**Table 2:** NMR spectroscopic data for meridianin H (**12**) in DMSO-*d*_6_.

	12		
Position	*δ*_C_^a^	*δ*_H_^b^	HMBC
1		12.14 (1H, s)	
2	130.0, CH	8.34 (1H, s)	3, 3a, 7a
3	114.7, C		
3a	116.3, C		
4	148.7, C		
4-OH		15.07 (1H, s)	3a, 4, 5
5	99.1, C		
6	128.6, CH	7.42 (1H, s)	4, 5, 7, 7a
7	93.0, C		
7a	136.1, C		
1′	-	-	-
2′	161.4, C		
2′-NH_2_		6.91 (2H, brs)	
3′	-	-	-
4′	159.3, C		
5′	104.6, CH	7.23 (1H, d, 5)	3, 4′, 6′
6′	159.4, CH	8.17 (1H, d, 5)	4′, 5′

^a^ 125 MHz, multiplicity from HSQC; ^b^ 500 MHz (integration, multiplicity, *J* (Hz)).

## Data Availability

Spectral data are contained within the article or Supplementary Material. These data are available in Table 1 and Table 2, and Supplementary Figures S1–S46.

## Supplementary Materials

The following are available online at https://www.mdpi.com/article/10.3390/md20030196/s1, Figure S1: Australindolone A (**1**) ^1^H NMR spectrum, Figure S2: Australindolone A (**1**) ^13^C NMR spectrum, Figure S3: Australindolone A (**1**) COSY NMR spectrum, Figure S4: Australindolone A (**1**) HMBC NMR spectrum, Figure S5: Australindolone A (**1**) HSQC NMR spectrum, Figure S6: Australindolone A (**1**) HRESIMS, Figure S7: Australindolone B (**2**) ^1^H NMR spectrum, Figure S8: Australindolone B (**2**) ^13^C NMR spectrum, Figure S9: Australindolone B (**2**) COSY NMR spectrum, Figure S10: Australindolone B (**2**) HMBC NMR spectrum, Figure S11: Australindolone B (**2**) HSQC NMR spectrum, Figure S12: Australindolone B (**2**) HRESIMS, Figure S13: Australindolone C (**3**) ^1^H NMR spectrum, Figure S14: Australindolone C (**3**) ^13^C NMR spectrum, Figure S15: Australindolone C (**3**) COSY NMR spectrum, Figure S16: Australindolone C (**3**) HMBC NMR spectrum, Figure S17: Australindolone C (**3**) HSQC NMR spectrum, Figure S18: Australindolone D (**4**) HRESIMS, Figure S19: Australindolone D (**4**) ^1^H NMR spectrum, Figure S20: Australindolone D (**4**) ^13^C NMR spectrum, Figure S21: Australindolone D (**4**) COSY NMR spectrum, Figure S22: Australindolone D (**4**) HMBC NMR spectrum, Figure S23: Australindolone D (**4**) HSQC NMR spectrum, Figure S24: Australindolone D (**4**) HRESIMS, Figure S25: Meridianin A (**5**) ^1^H NMR spectrum, Figure S26: Meridianin A (**5**) ^13^C NMR spectrum, Figure S27: Meridianin A (**5**) HRSEIMS, Figure S28: Meridianin B (**6**) ^1^H NMR spectrum, Figure S29: Meridianin B (**6**) HRESIMS, Figure S30: Meridianin C (**7**) ^1^H NMR spectrum, Figure S31: Meridianin C (**7**) ^13^C NMR spectrum, Figure S32: Meridianin C (**7**) HRESIMS, Figure S33: Meridianin D (8) ^1^H NMR spectrum, Figure S34: Meridianin D (**8**) HRESIMS, Figure S35: Meridianin E (**9**) ^1^H NMR spectrum, Figure S36: Meridianin E (**9**) HRESIMS, Figure S37: Meridianin F (**10**) ^1^H NMR spectrum, Figure S38: Meridianin F (**10**) HRESIMS, Figure S39: Meridianin G (**11**) ^1^H NMR spectrum, Figure S40: Meridianin G (**11**) HRESIMS, Figure S41: Meridianin H (**12**) ^1^H NMR spectrum, Figure S42: Meridianin H (**12**) ^13^C NMR spectrum, Figure S43: Meridianin H (**12**) COSY NMR spectrum, Figure S44: Meridianin H (**12**) HMBC NMR spectrum, Figure S45: Meridianin H (**12**) HSQC NMR spectrum, Figure S46: Meridianin H (**12**) HRESIMS, Table S1: Crystal data and structure refinement for australindolone B (**2**).
