# Supplementary material for: Australindolones, New Aminopyrimidine Substituted Indolone Alkaloids from an Antarctic Tunicate Synoicum sp
Source: Mar Drugs. 2022 Mar 8;20(3):196. doi: 10.3390/md20030196 (PMC8949045; doi:10.3390/md20030196)
Supplement: Supplementary file 1 [file marinedrugs-20-00196-s001.zip › marinedrugs-1612108-supplementary.pdf]

Australindolones, new aminopyrimidine substituted indolone alkaloids from an Antarctic tunicate *Synoicum* sp.

Sofia Kokkaliari,[1] Kim Pham,[1] Nargess Shahbazi,[2,5] Laurent Calcul,[1] Lukasz Wojtas,[1] Nerida G. Wilson,[3] Alexander D. Crawford[2,4] and Bill J. Baker[1,4,*]

[1] Department of Chemistry, University of South Florida, 4202 E. Fowler Ave., CHE205, Tampa, Florida 33620, USA

[2] Department of Preclinical Sciences and Pathology, Norwegian University of Life Sciences (NMBU), Ås, Norway

[3] Research & Collections, Western Australia Museum, 49 Kew Street, Welshpool 6106 and School of Biological Sciences, University of Western Australia, 35 Stirling Highway, Crawley 6009, Western Australia, Australia

[4] Institute for Arctic and Antarctic Biodiscovery, Medford, Oregon, USA

[5] Current address: Institute of Molecular Biology, University of Innsbruck, Innsbruck, Austria

* Correspondence: bjbaker@usf.edu

Data	Page
**Figure S1.** Australindolone A (**1**) ^{1}H NMR spectrum (500 MHz, DMSO-d_6)	2
**Figure S2.** Australindolone A (**1**) ^{13}C NMR spectrum (125 MHz, DMSO-d_6)	3
**Figure S3.** Australindolone A (**1**) COSY NMR spectrum (500 MHz, DMSO-d_6)	3
**Figure S4.** Australindolone A (**1**) HMBC NMR spectrum (500 MHz, DMSO-d_6)	4
**Figure S5.** Australindolone A (**1**) HSQC NMR spectrum (500 MHz, DMSO-d_6)	4
**Figure S6.** Australindolone A (**1**) HRESIMS(+)	5
**Figure S7.** Australindolone B (**2**) ^{1}H NMR spectrum (500 MHz, DMSO-d_6)	5
**Figure S8.** Australindolone B (**2**) ^{13}C NMR spectrum (125 MHz, DMSO-d_6)	6
**Figure S9.** Australindolone B (**2**) COSY NMR spectrum (500 MHz, DMSO-d_6)	6
**Figure S10.** Australindolone B (**2**) HMBC NMR spectrum (500 MHz, DMSO-d_6)	7
**Figure S11.** Australindolone B (**2**) HSQC NMR spectrum (500 MHz, DMSO-d_6)	7
**Figure S12.** Australindolone B (**2**) HRESIMS(+)	8
**Figure S13.** Australindolone C (**3**) ^{1}H NMR spectrum (500 MHz, DMSO-d_6)	8
**Figure S14.** Australindolone C (**3**) ^{13}C NMR spectrum (125 MHz, DMSO-d_6)	9
**Figure S15.** Australindolone C (**3**) COSY NMR spectrum (500 MHz, DMSO-d_6)	9
**Figure S16.** Australindolone C (**3**) HMBC NMR spectrum (500 MHz, DMSO-d_6)	10
**Figure S17.** Australindolone C (**3**) HSQC NMR spectrum (500 MHz, DMSO-d_6)	10
**Figure S18.** Australindolone D (**4**) HRESIMS(+)	11
**Figure S19.** Australindolone D (**4**) ^{1}H NMR spectrum (500 MHz, DMSO-d_6)	11
**Figure S20.** Australindolone D (**4**) ^{13}C NMR spectrum (125 MHz, DMSO-d_6)	12
**Figure S21.** Australindolone D (**4**) COSY NMR spectrum (500 MHz, DMSO-d_6)	12
**Figure S22.** Australindolone D (**4**) HMBC NMR spectrum (500 MHz, DMSO-d_6)	13
**Figure S23.** Australindolone D (**4**) HSQC NMR spectrum (500 MHz, DMSO-d_6)	13
**Figure S24.** Australindolone D (**4**) HRESIMS(+)	14
**Figure S25.** Meridianin A (**5**) ^{1}H NMR spectrum (400 MHz, DMSO-d_6)	14
**Figure S26.** Meridianin A (**5**) ^{13}C NMR spectrum (100 MHz, DMSO d_6)	15
**Figure S27.** Meridianin A (**5**) HRSEIMS(+)	15
**Figure S28.** Meridianin B (**6**) ^{1}H NMR spectrum (400 MHz, DMSO-d_6)	16
**Figure S29.** Meridianin B (**6**) HRESIMS	16
**Figure S30.** Meridianin C (**7**) ^{1}H NMR spectrum (400 MHz, DMSO-d_6)	17
**Figure S31.** Meridianin C (**7**) ^{13}C NMR spectrum (100 MHz, DMSO-d_6)	17

Figure S32. Meridianin C (7) HRESIMS(+) 18
Figure S33. Meridianin D (**8**) ^{1}H NMR spectrum (400 MHz, DMSO-d_6) 18
Figure S34. Meridianin D (**8**) HRESIMS(+) 19
Figure S35. Meridianin E (**9**) ^{1}H NMR spectrum (400 MHz, DMSO-d_6) 19
Figure S36. Meridianin E (**9**) HRESIMS(+) 20
Figure S37. Meridianin F (**10**) ^{1}H NMR spectrum (400 MHz, CD_3OD) 20
Figure S38. Meridianin F (**10**) HRESIMS(+) 21
Figure S39. Meridianin G (**11**) ^{1}H NMR spectrum (400 MHz, CD_3OD) 21
Figure S40. Meridianin G (**11**) HRESIMS(+) 22
Figure S41. Meridianin H (**12**) ^{1}H NMR spectrum (500 MHz, DMSO-d_6) 22
Figure S42. Meridianin H (**12**) ^{13}C NMR spectrum (125 MHz, DMSO-d_6) 23
Figure S43. Meridianin H (**12**) COSY NMR spectrum (500 MHz, DMSO-d_6) 23
Figure S44. Meridianin H (**12**) HMBC NMR spectrum (500 MHz, DMSO-d_6) 24
Figure S45. Meridianin H (**12**) HSQC NMR spectrum (500 MHz, DMSO-d_6) 24
Figure S46. Meridianin H (**12**) HRESIMS(+) 25
Table S1. Crystal data and structure refinement for australindolone B (**2**) 26

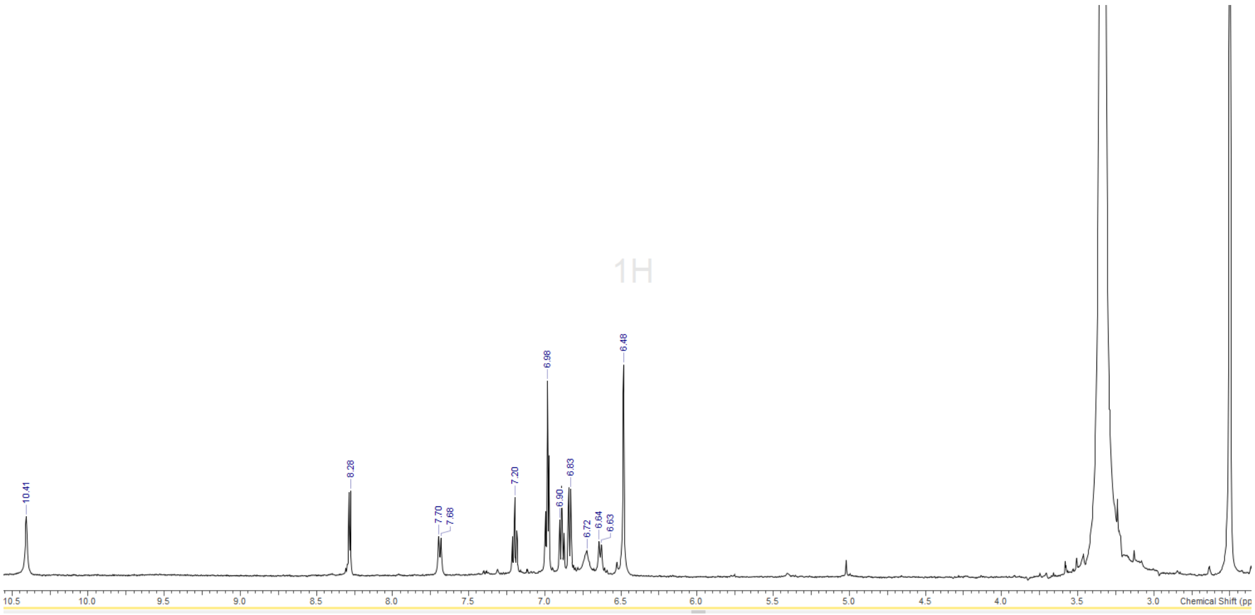

Figure S1. Australindolone A (**1**) ^{1}H NMR spectrum (500 MHz, DMSO-d_6)

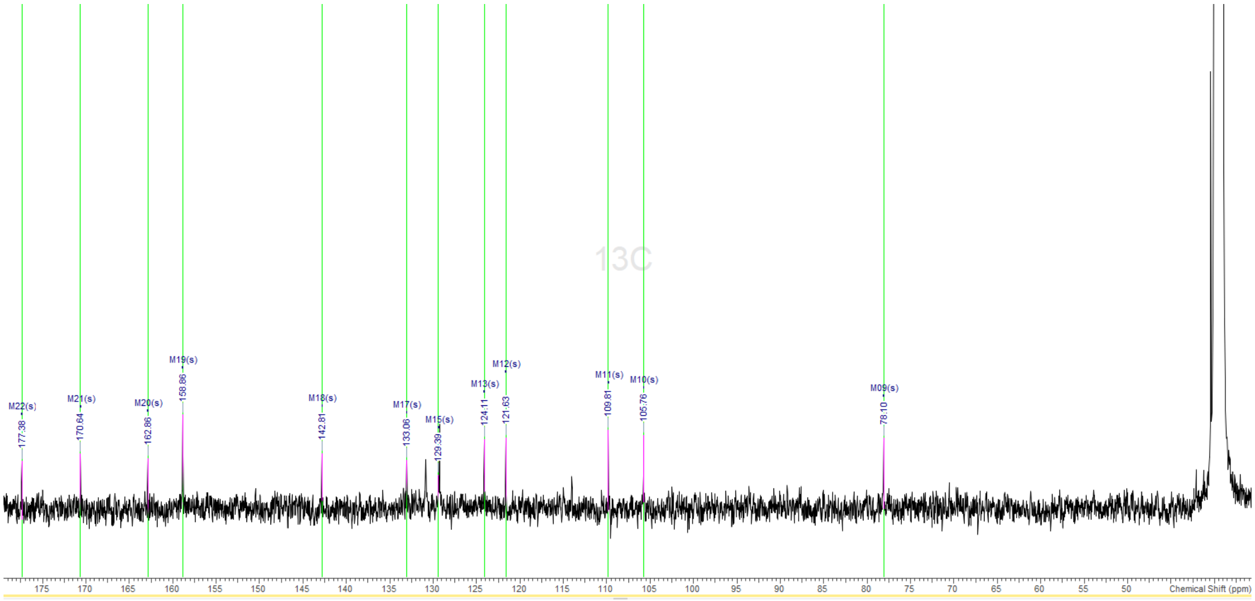

Figure S2. Australindolone A (**1**) ^{13}C NMR spectrum (125 MHz, DMSO-*d*$_6$)

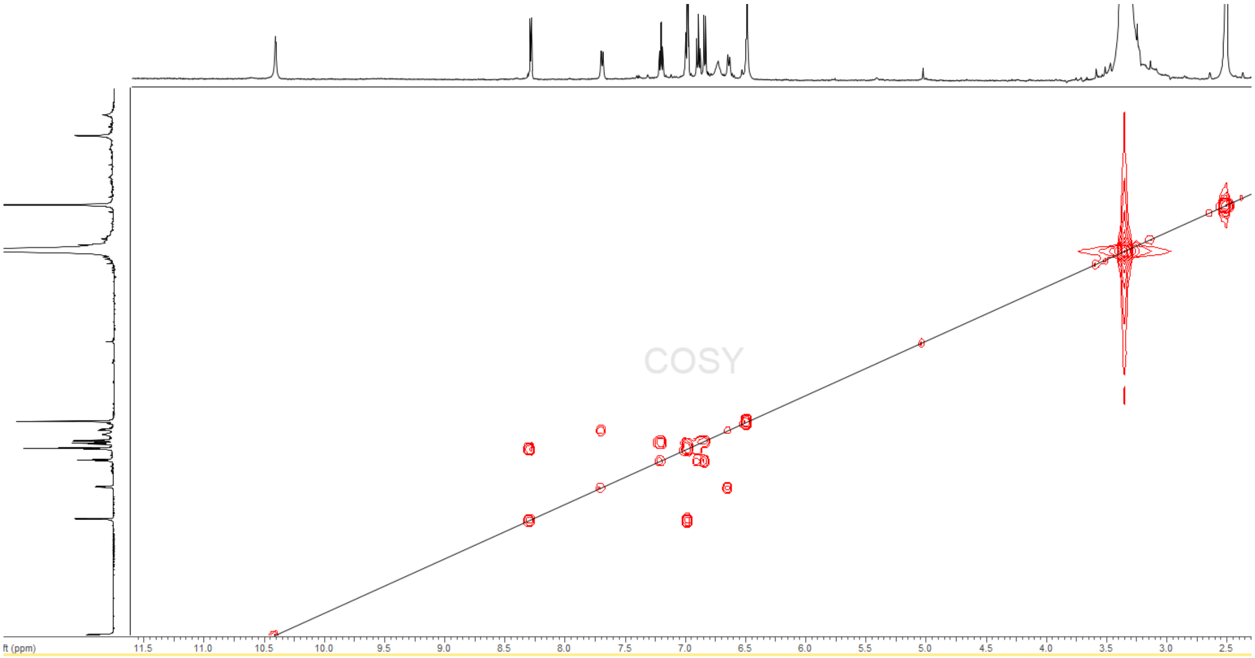

Figure S3. Australindolone A (**1**) COSY NMR spectrum (500 MHz, DMSO-*d*$_6$)

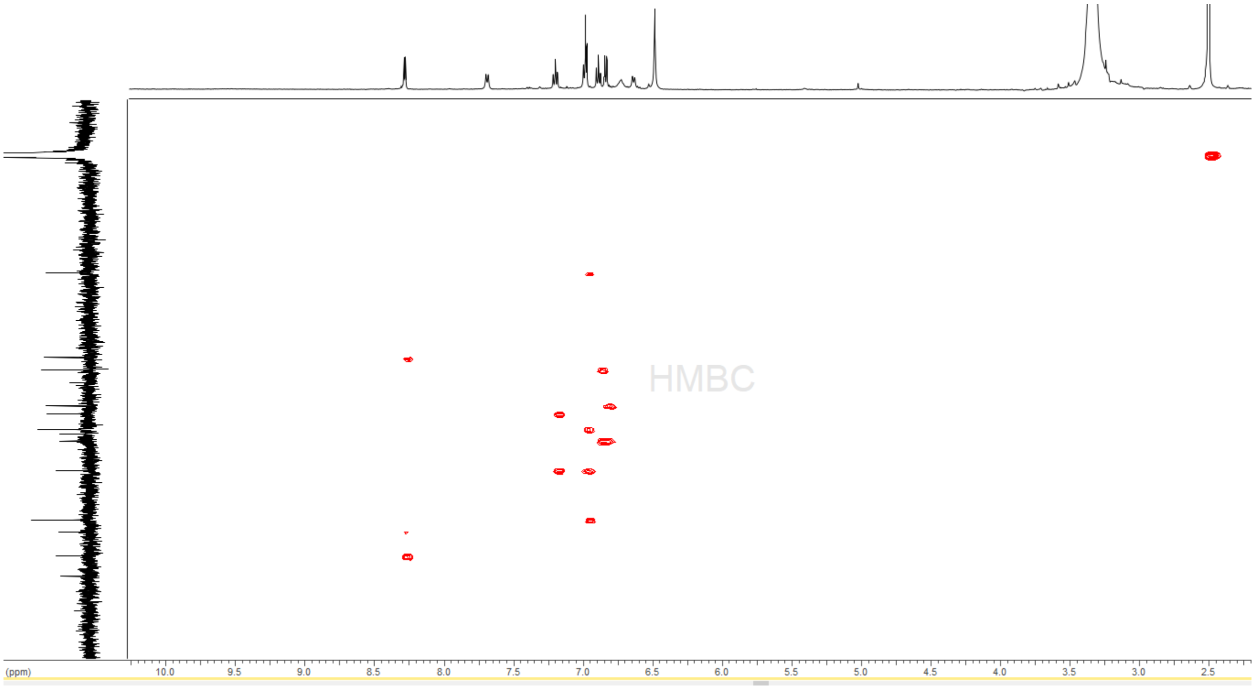

Figure S4. Australindolone A (**1**) HMBC NMR spectrum (500 MHz, DMSO-d_6)

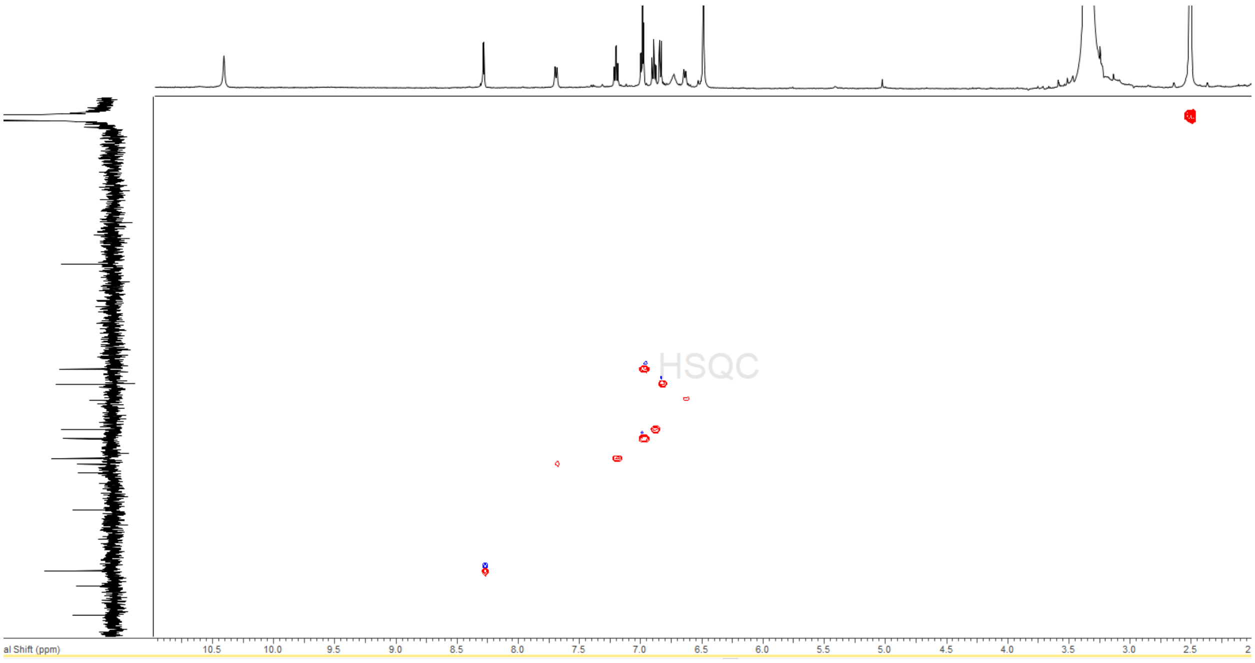

Figure S5. Australindolone A (**1**) HSQC NMR spectrum (500 MHz, DMSO-d_6)

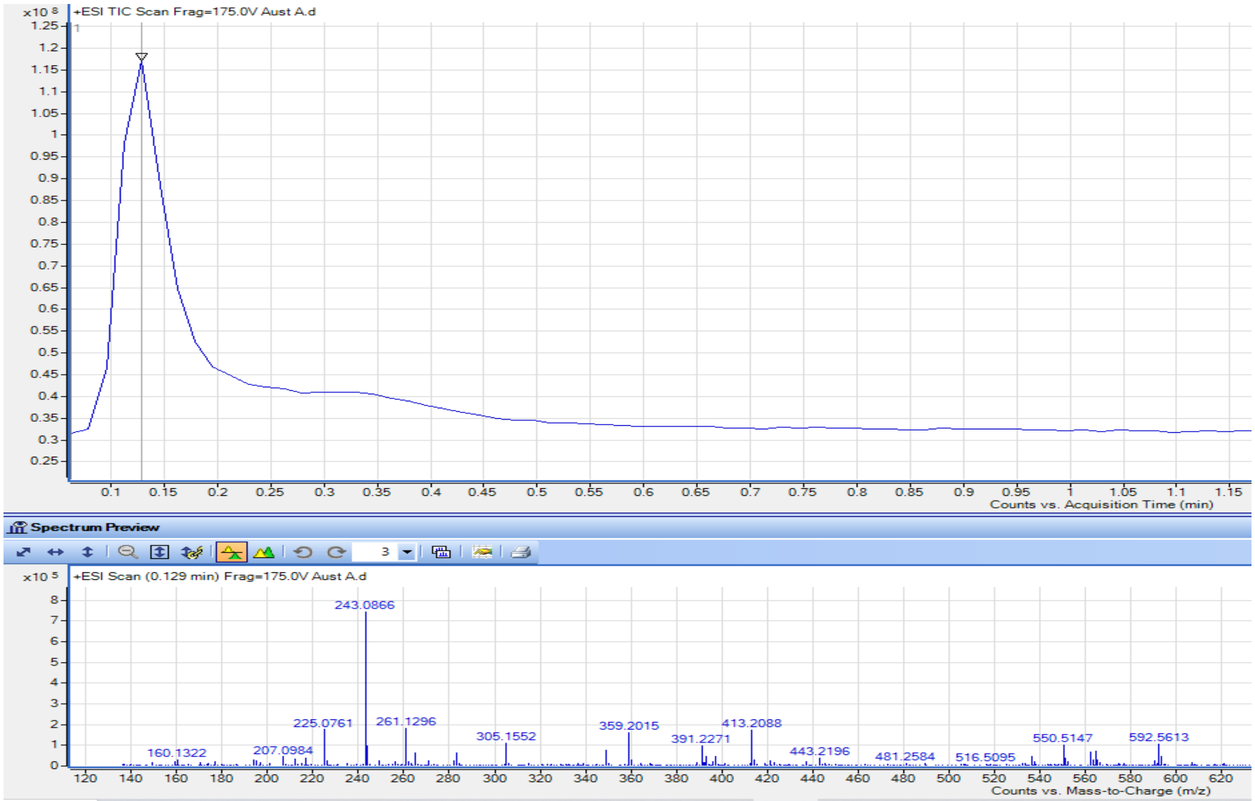

Figure S6. Australindolone A (**1**) HRESIMS(+)

.

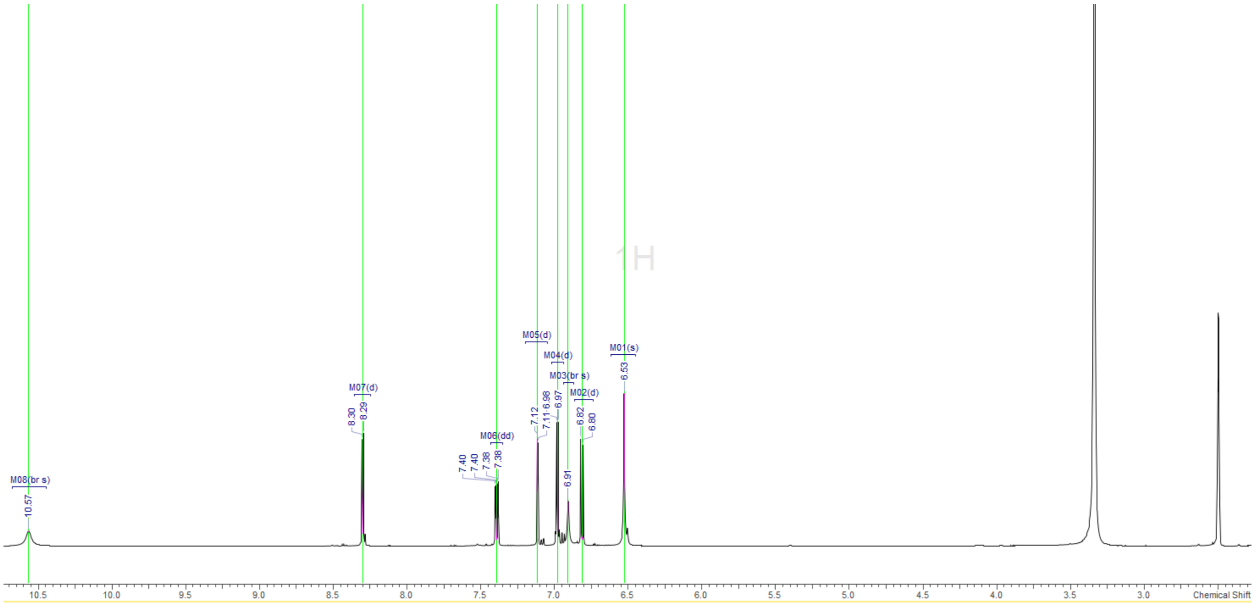

Figure S7. Australindolone B (**2**) ^{1}H NMR spectrum (500 MHz, DMSO-d_6)

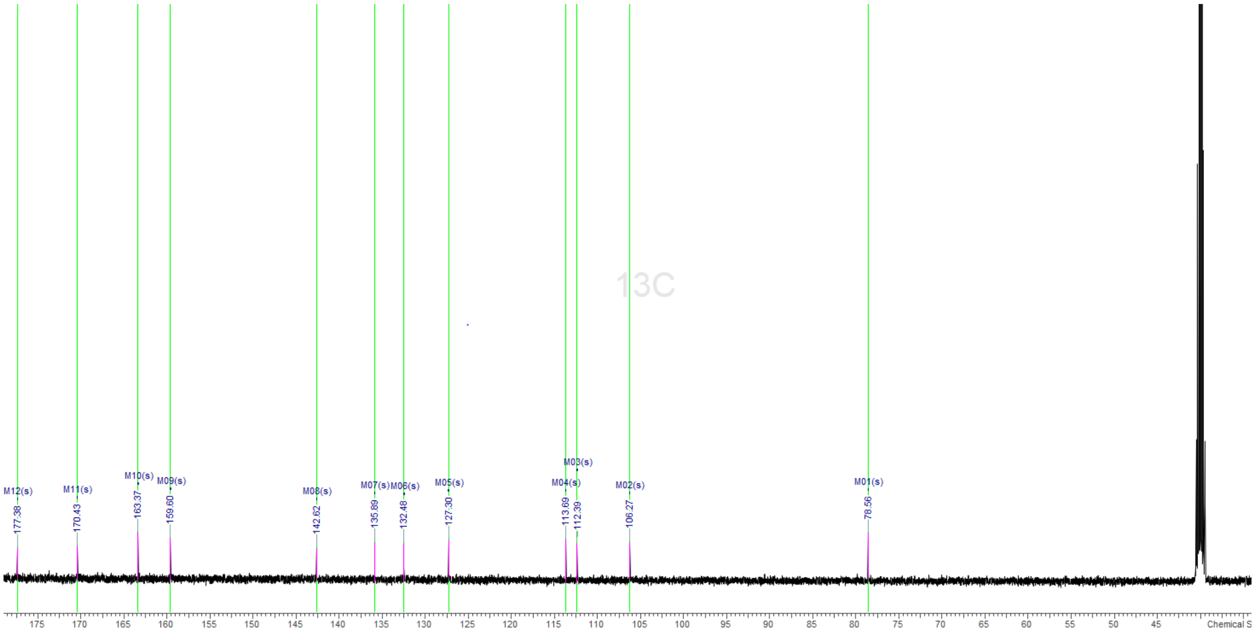

Figure S8. Australindolone B (**2**) ^{13}C NMR spectrum (125 MHz, DMSO-d_6)

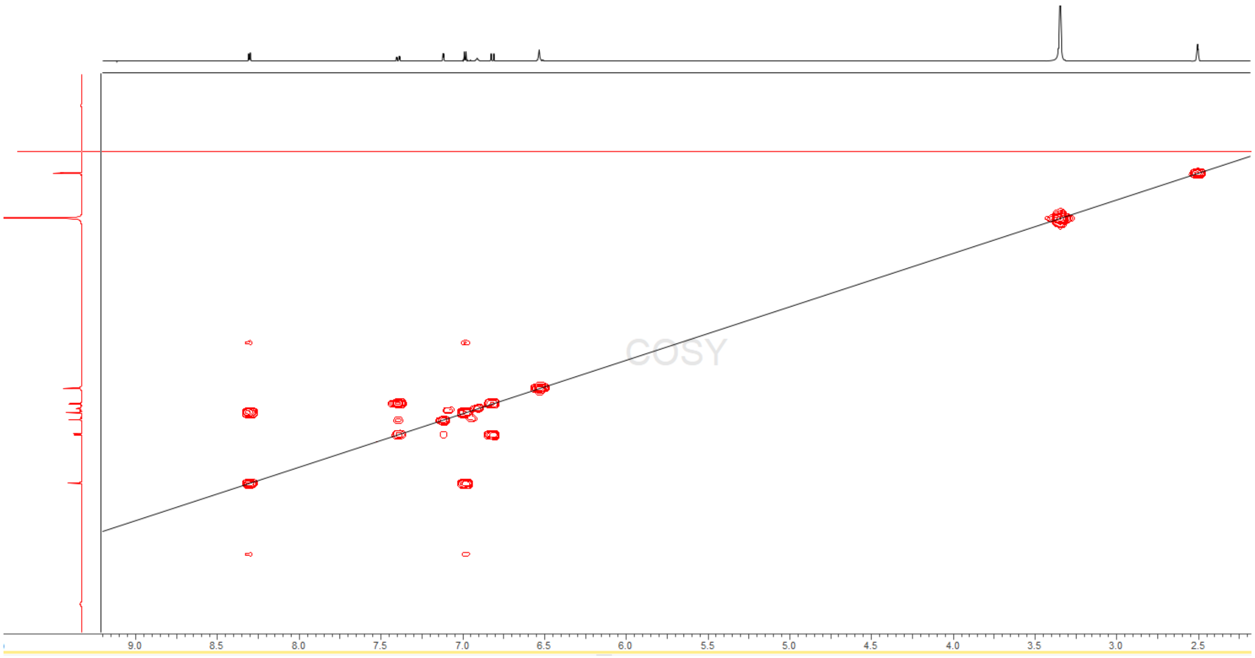

Figure S9. Australindolone B (**2**) COSY NMR spectrum (500 MHz, DMSO-d_6)

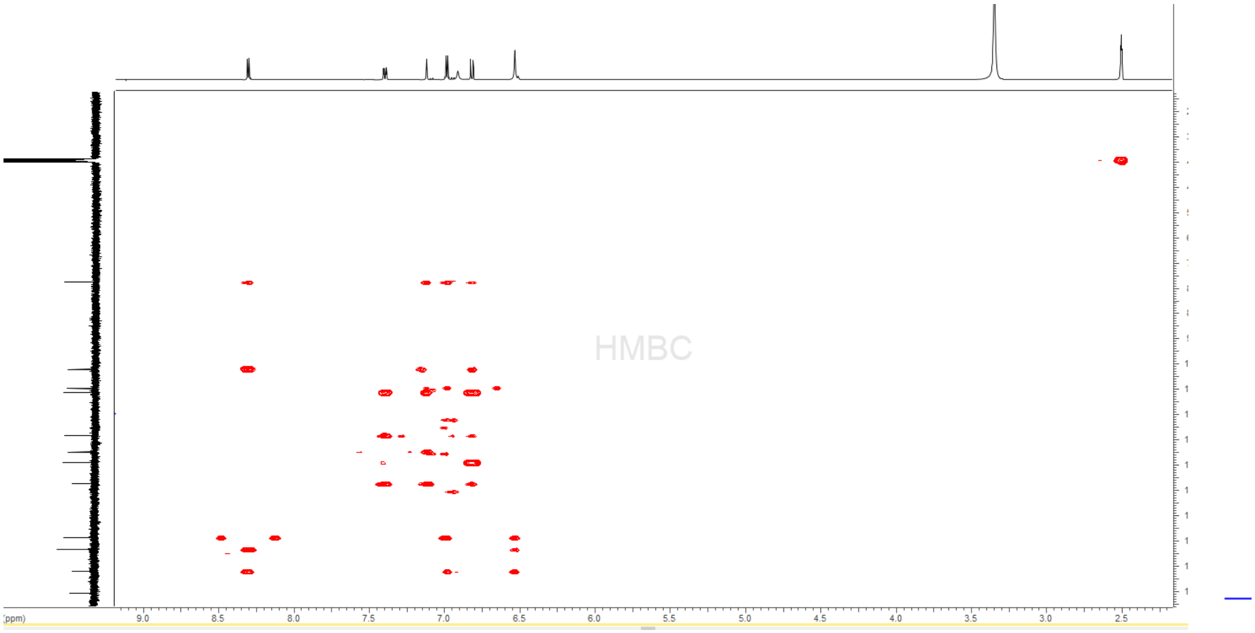

Figure S10. Australindolone B (**2**) HMBC NMR spectrum (500 MHz, DMSO-d_6)

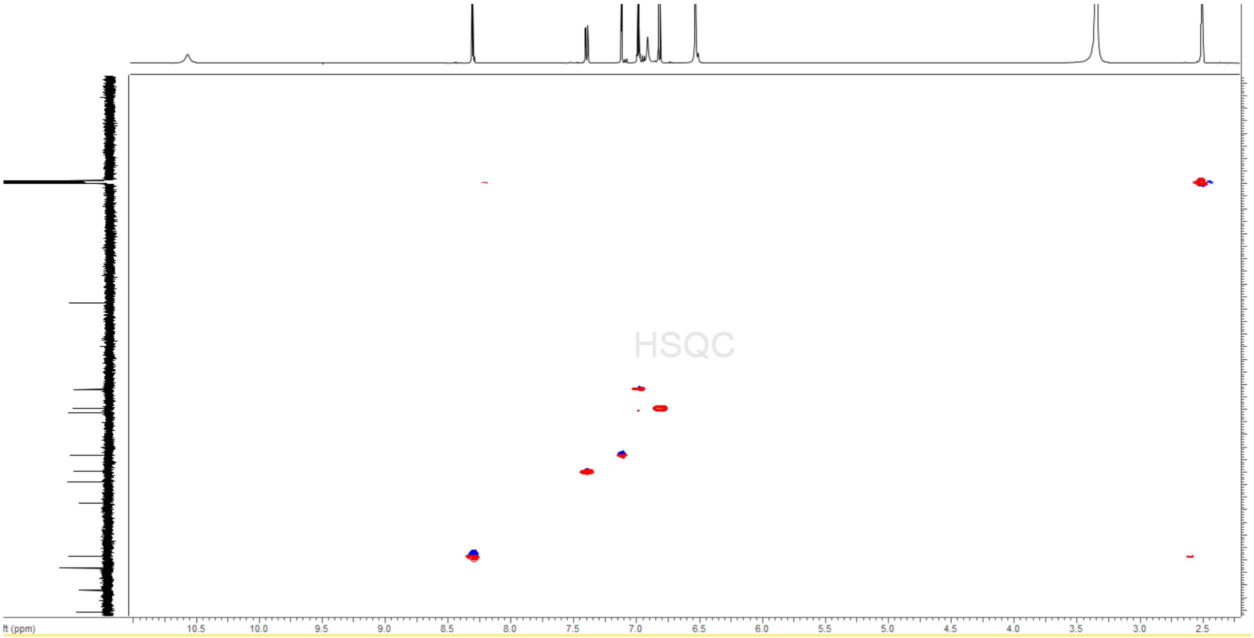

Figure S11. Australindolone B (**2**) HSQC NMR spectrum (500 MHz, DMSO-d_6)

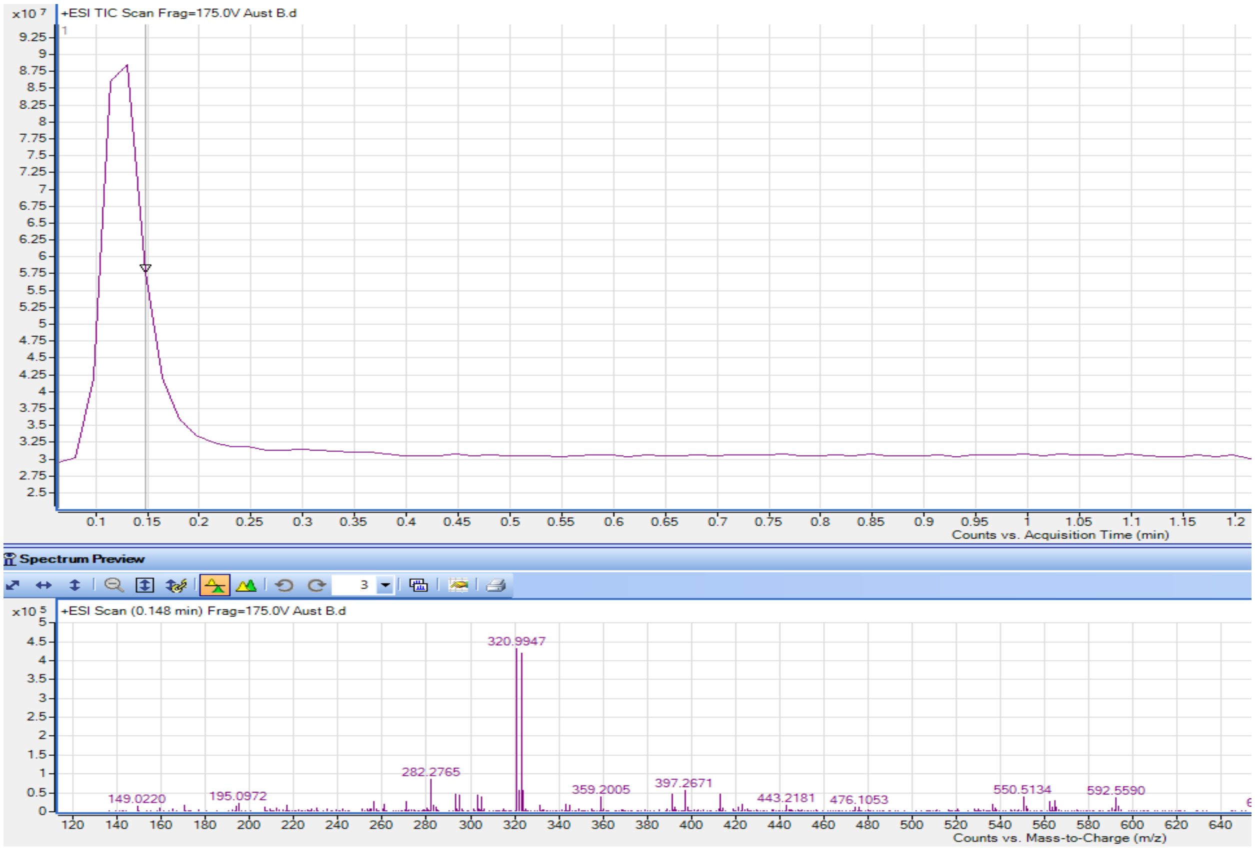

Figure S12. Australindolone B (**2**) HRESIMS(+)

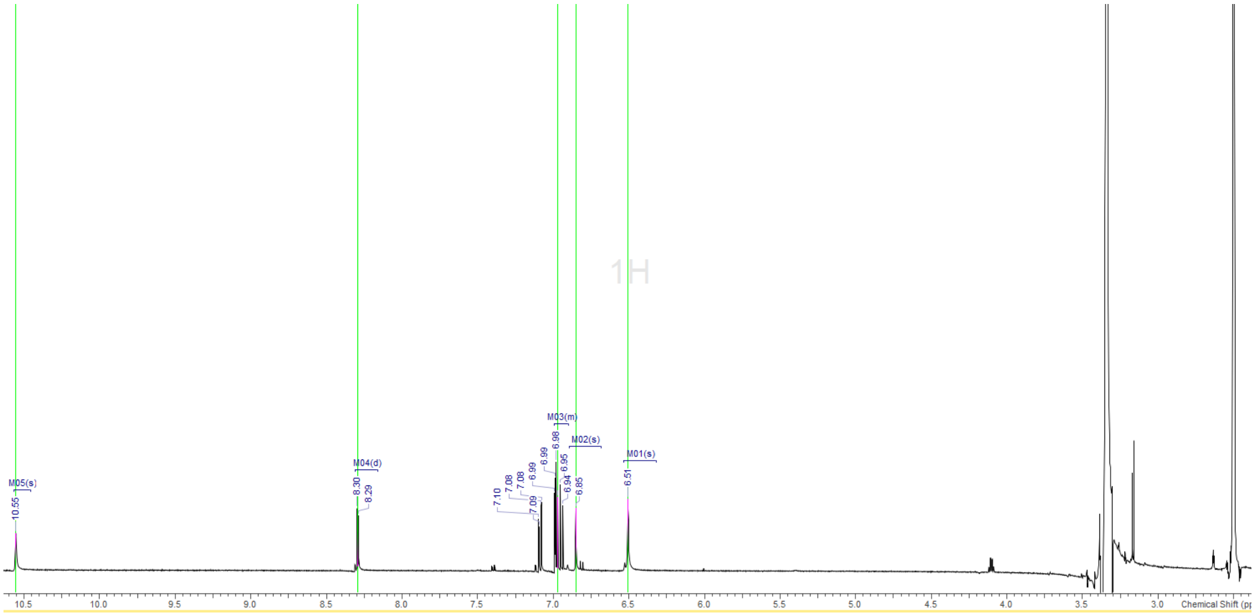

Figure S13. Australindolone C (**3**) ^{1}H NMR spectrum (500 MHz, DMSO-d_6)

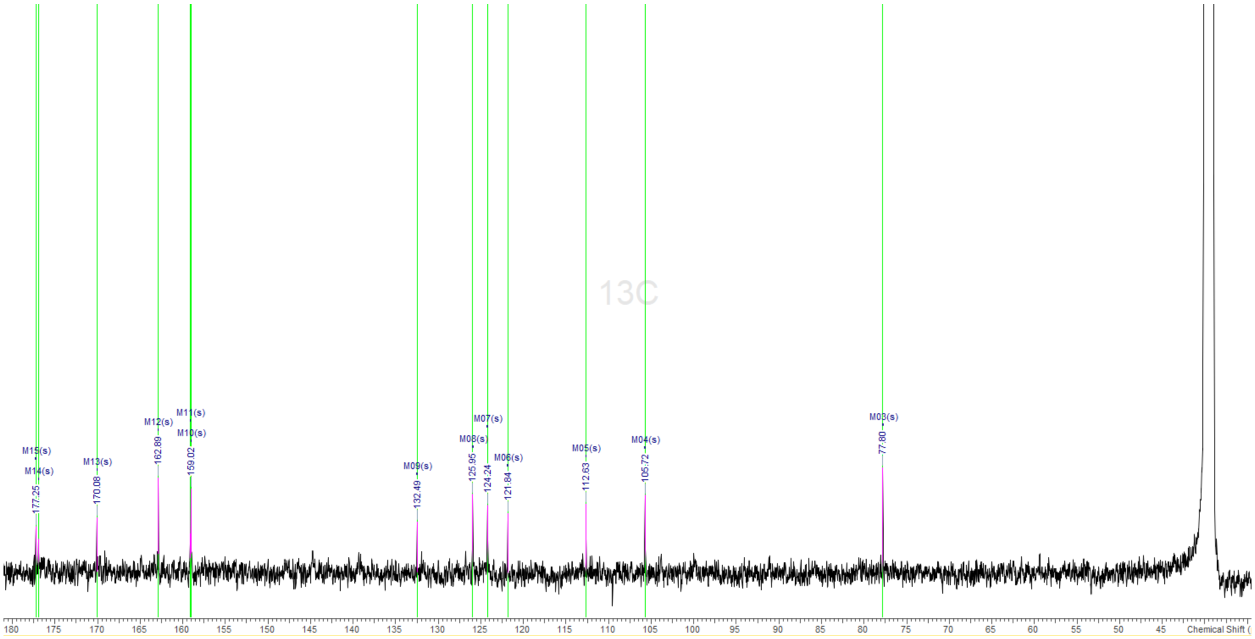

Figure S14. Australindolone C (**3**) ^{13}C NMR spectrum (125 MHz, DMSO-d_6)

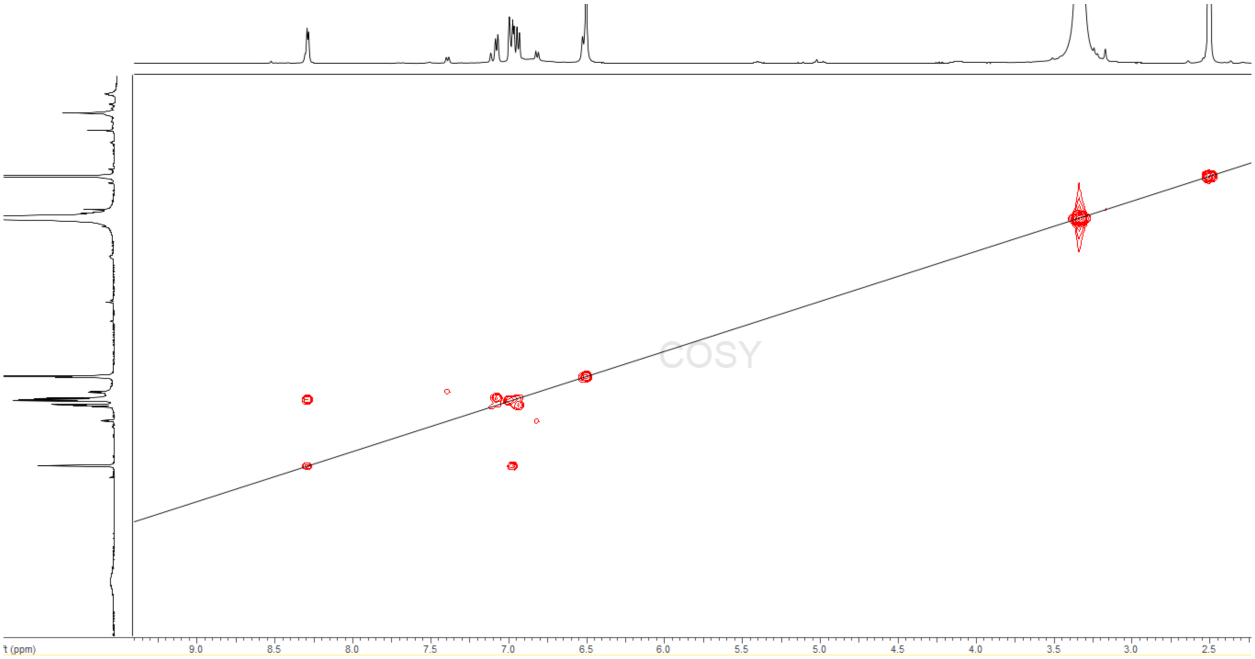

Figure S15. Australindolone C (**3**) COSY NMR spectrum (500 MHz, DMSO-d_6)

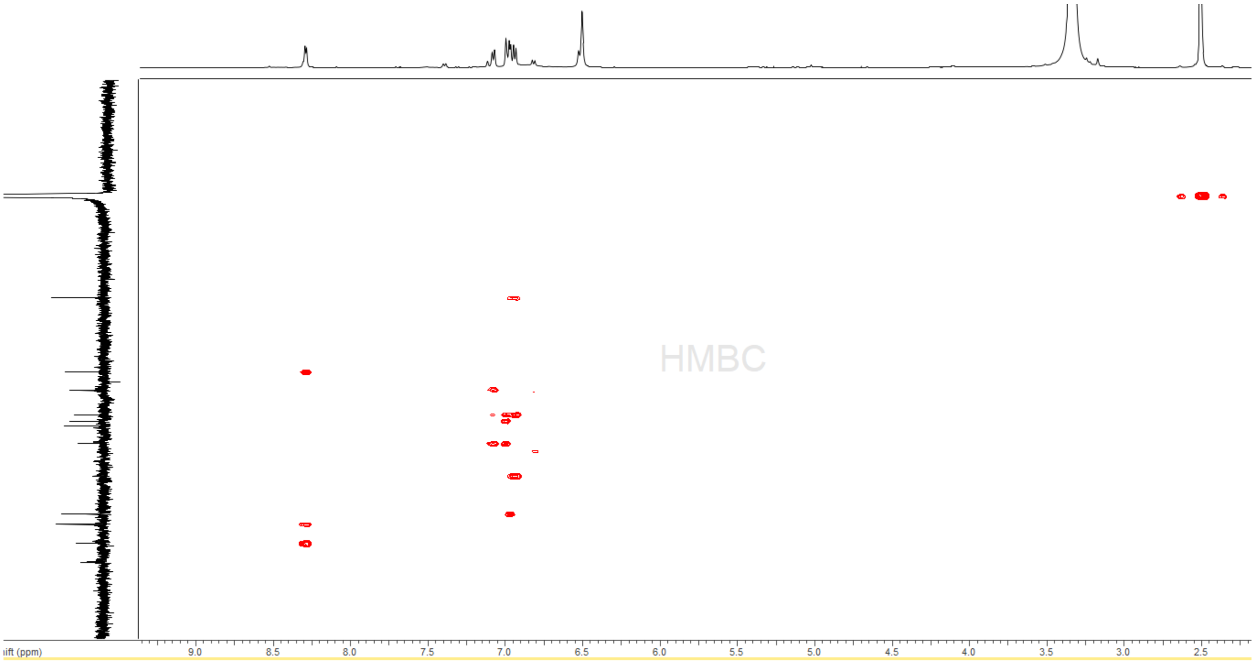

Figure S16. Australindolone C (**3**) HMBC NMR spectrum (500 MHz, DMSO-d_6)

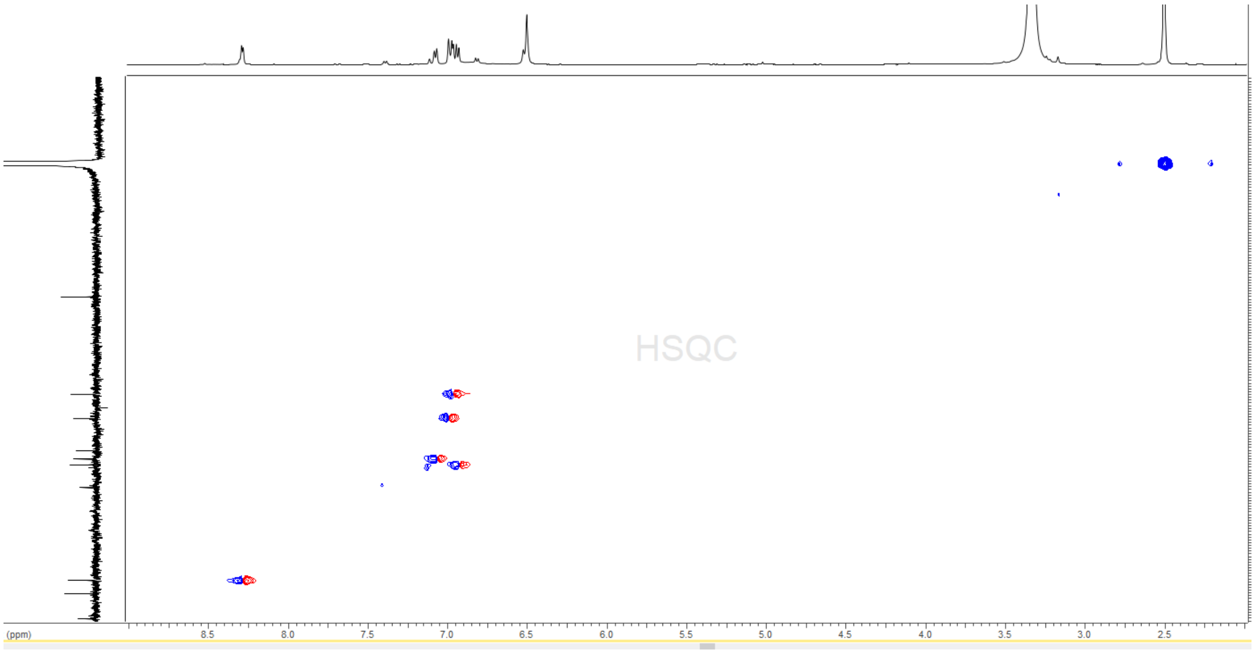

Figure S17. Australindolone C (**3**) HSQC NMR spectrum (500 MHz, DMSO-d_6)

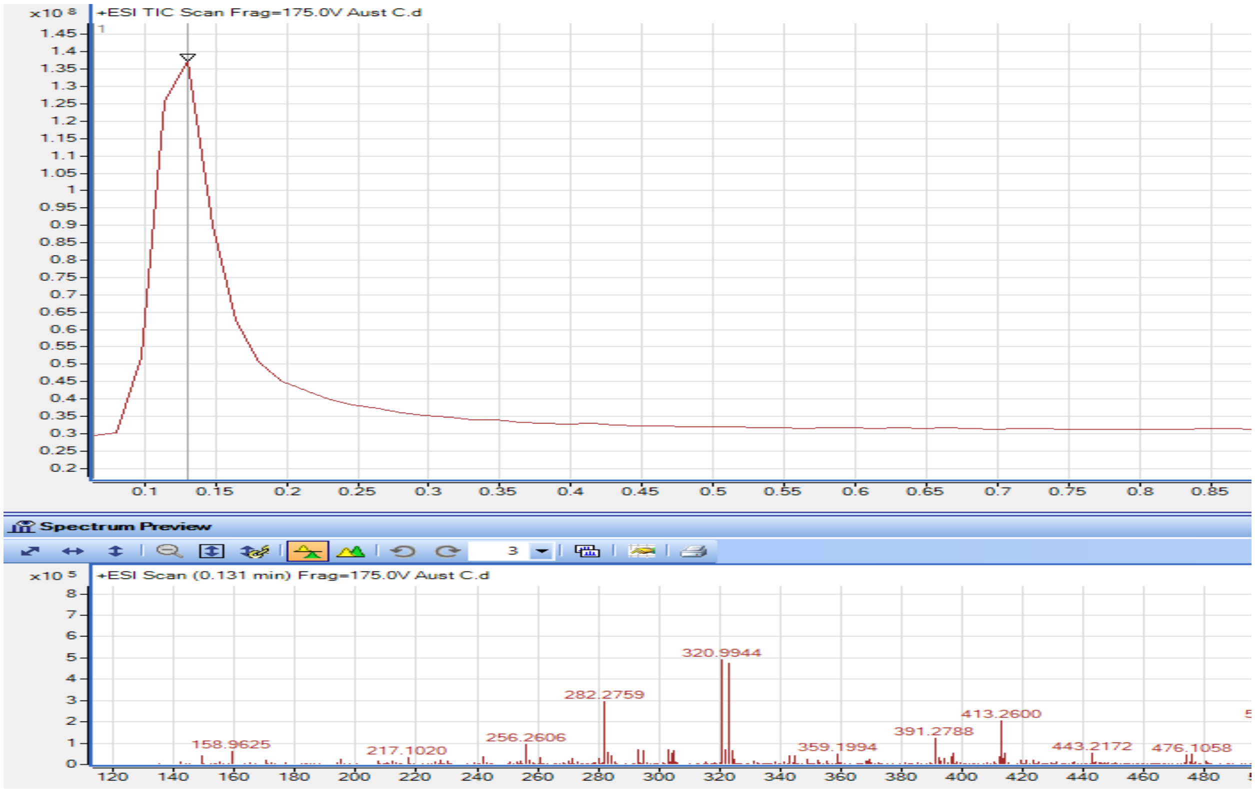

Figure S18. Australindolone C (**3**) HRESIMS(+)

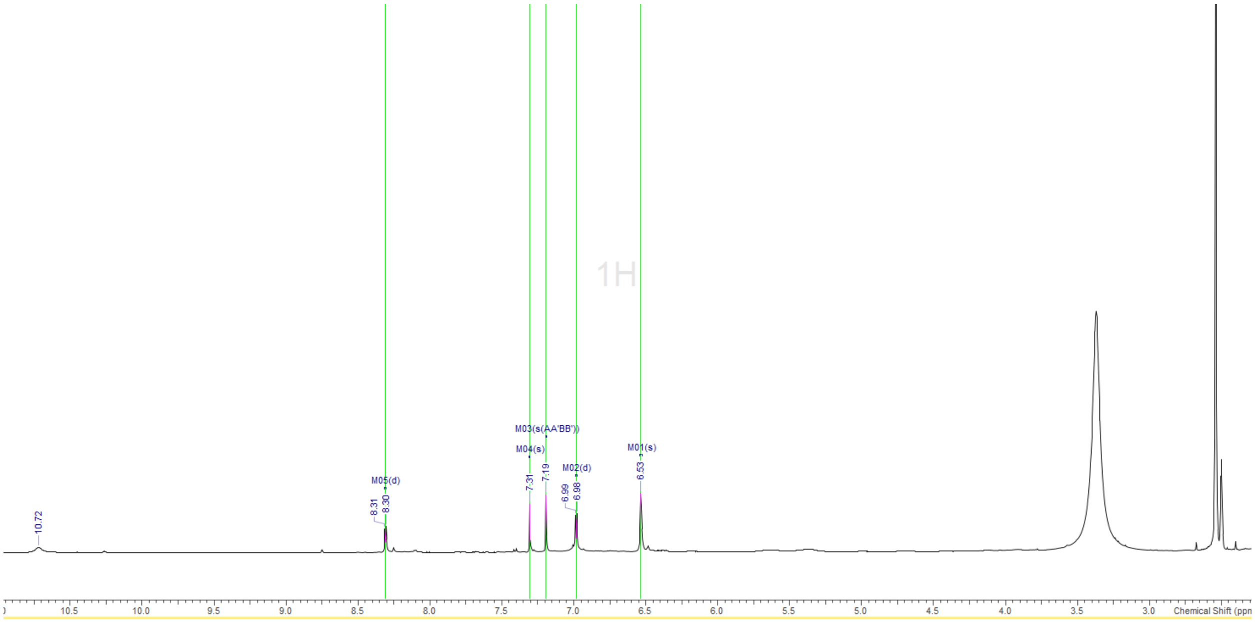

Figure S19. Australindolone D (**4**) ^{1}H NMR spectrum (500 MHz, DMSO-d_6)

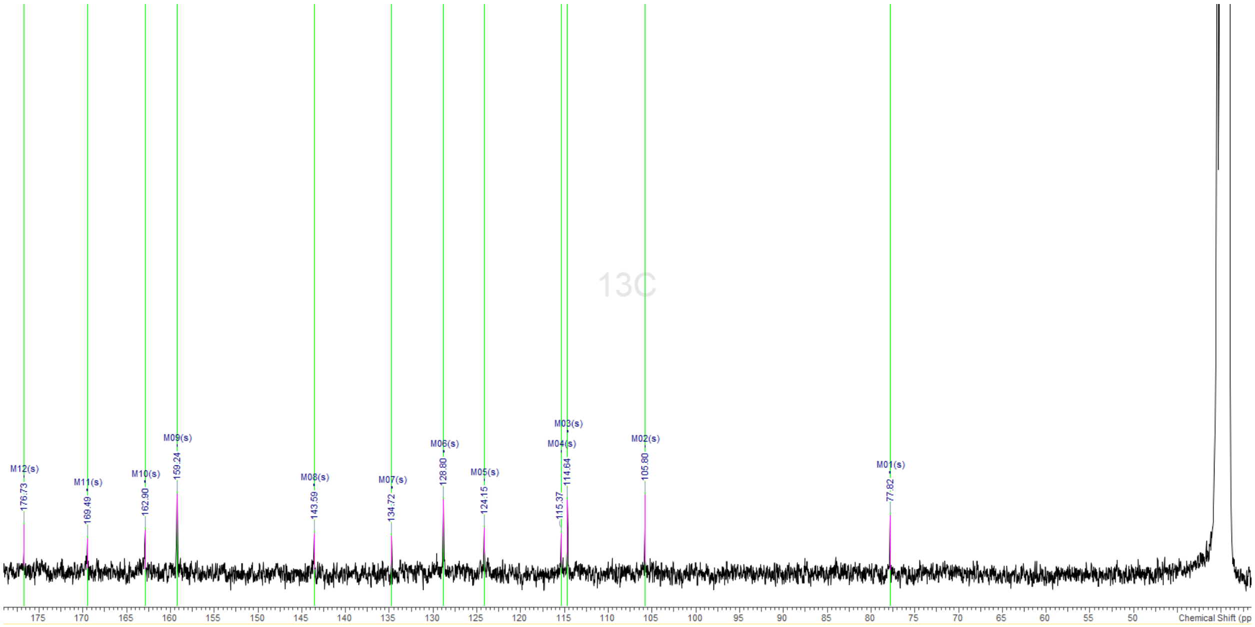

Figure S20. Australindolone D (**4**) ^{13}C NMR spectrum (125 MHz, DMSO-d_6)

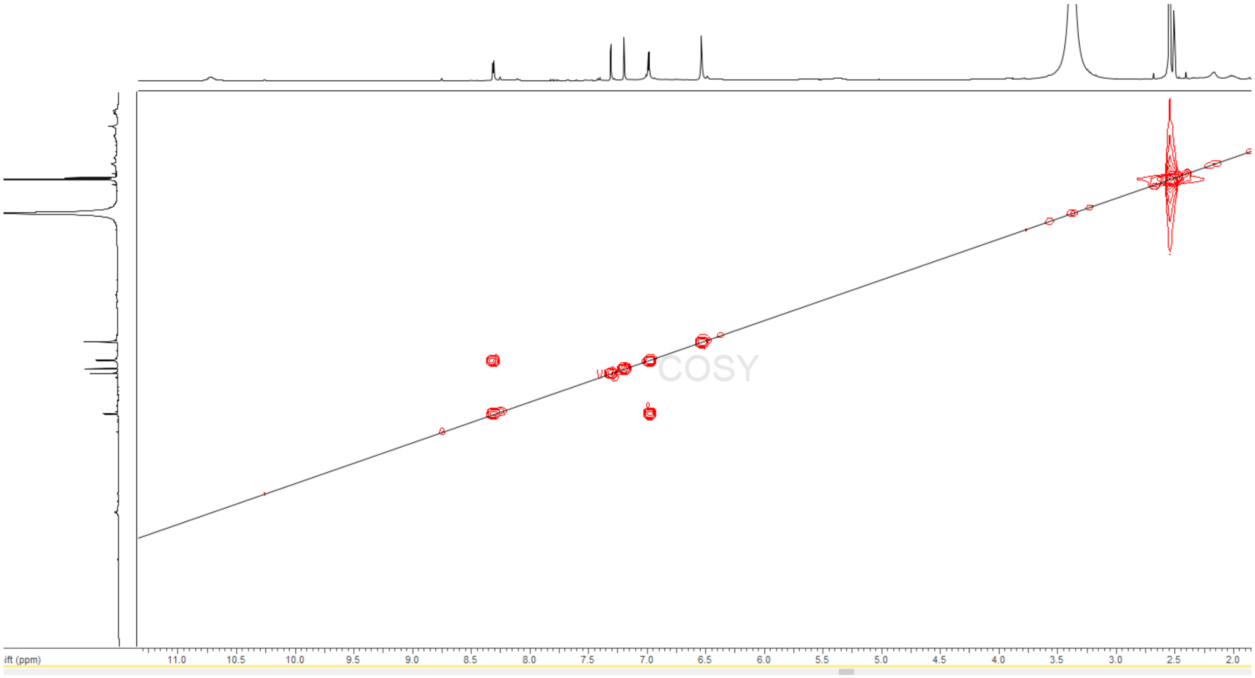

Figure S21. Australindolone D (**4**) COSY NMR spectrum (500 MHz, DMSO-d_6)

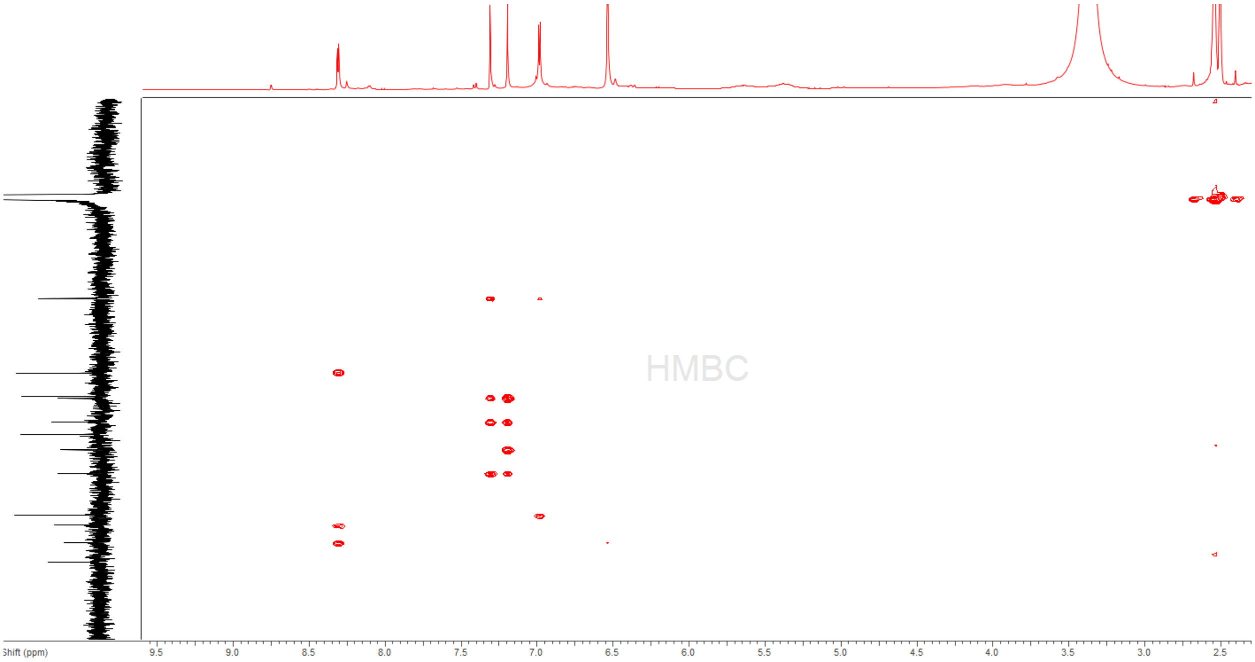

Figure S22. Australindolone D (**4**) HMBC NMR spectrum (500 MHz, DMSO-d_6)

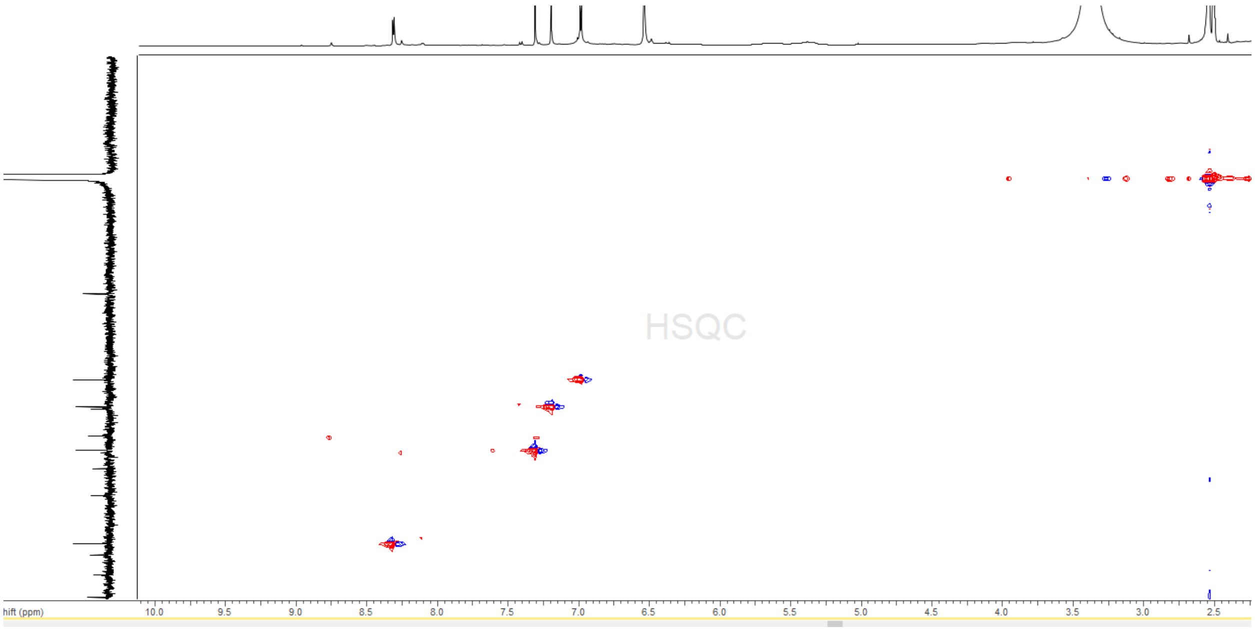

Figure S23. Australindolone D (**4**) HSQC NMR spectrum (500 MHz, DMSO-d_6)

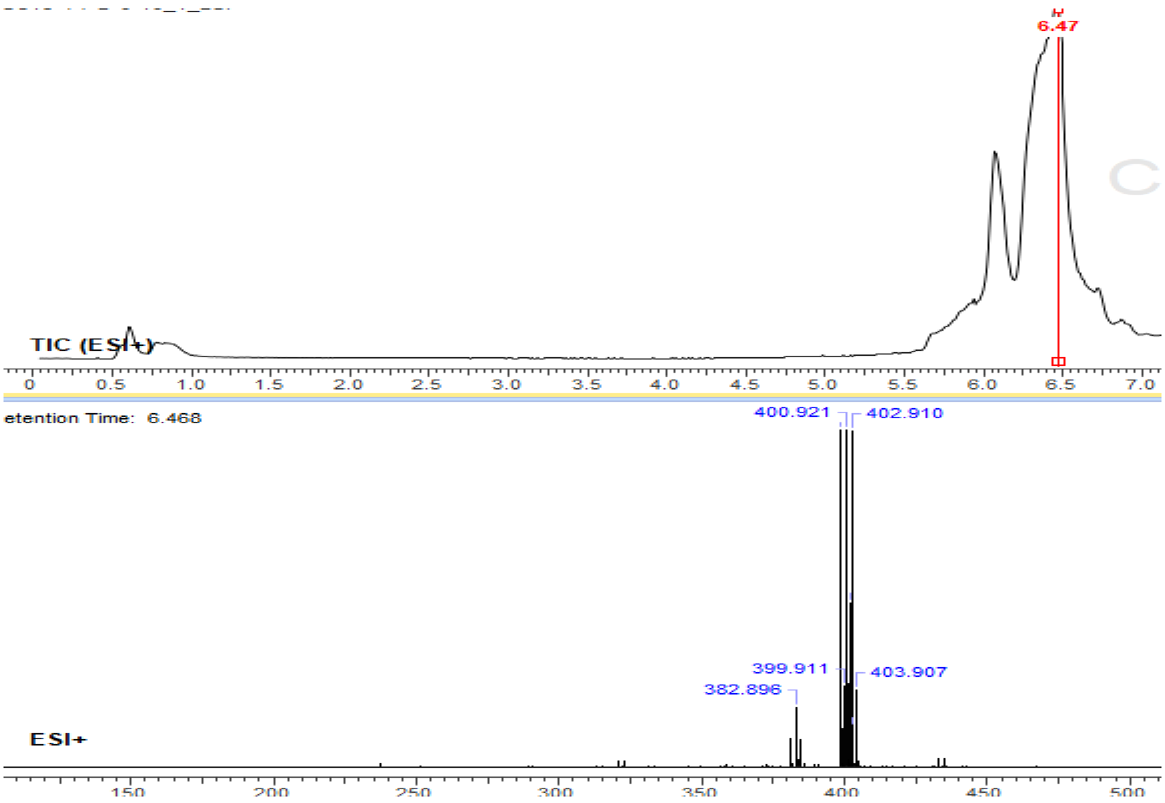

Figure S24. Australindolone D (**4**) HRESIMS(+)

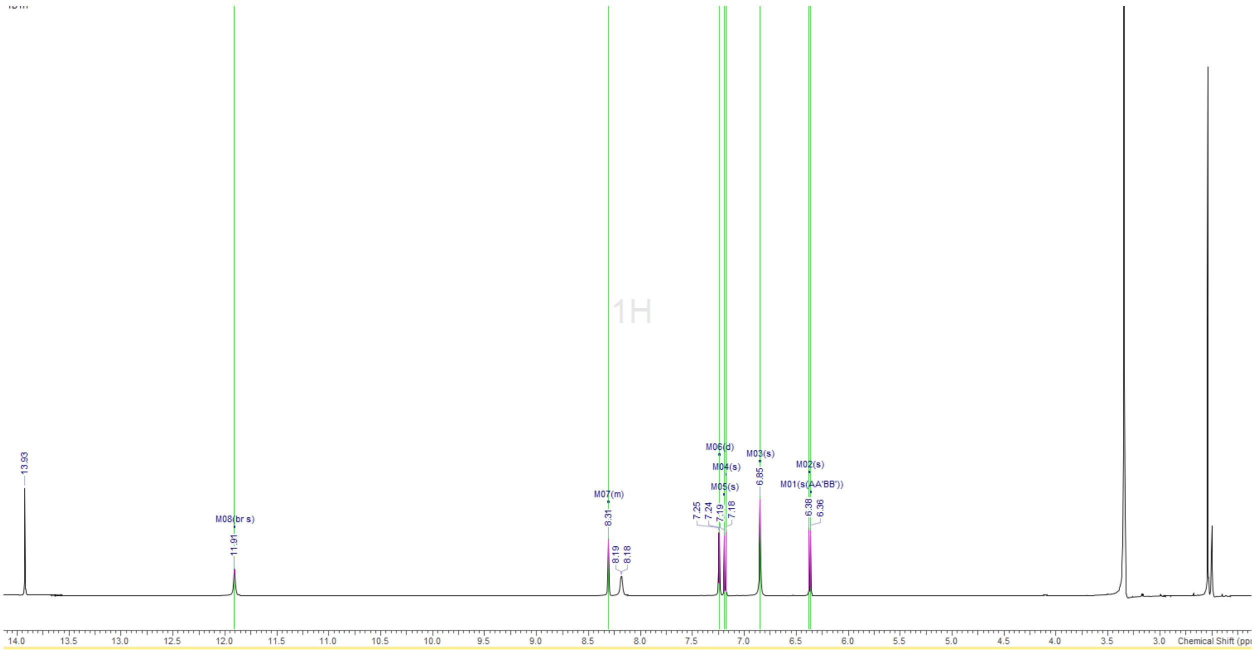

Figure S25. Meridianin A (**5**) ^{1}H NMR spectrum (400 MHz, DMSO-d_6)

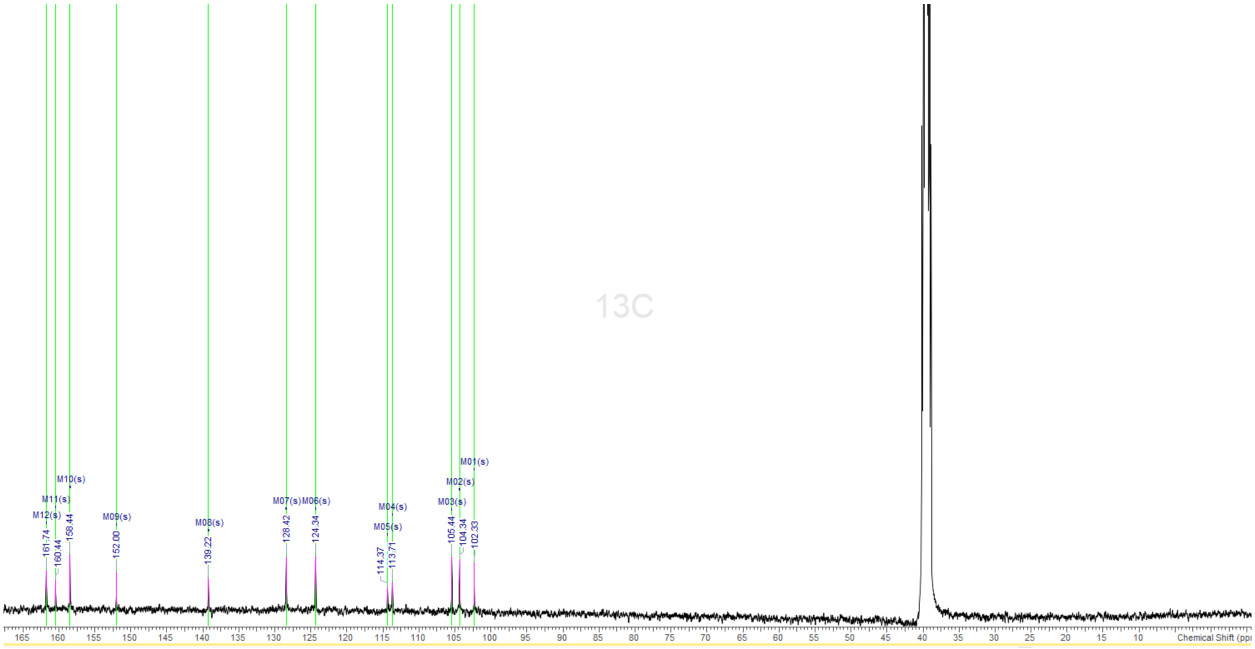

Figure S26. Meridianin A (**5**) ^{13}C NMR spectrum (100 MHz, DMSO d_6)

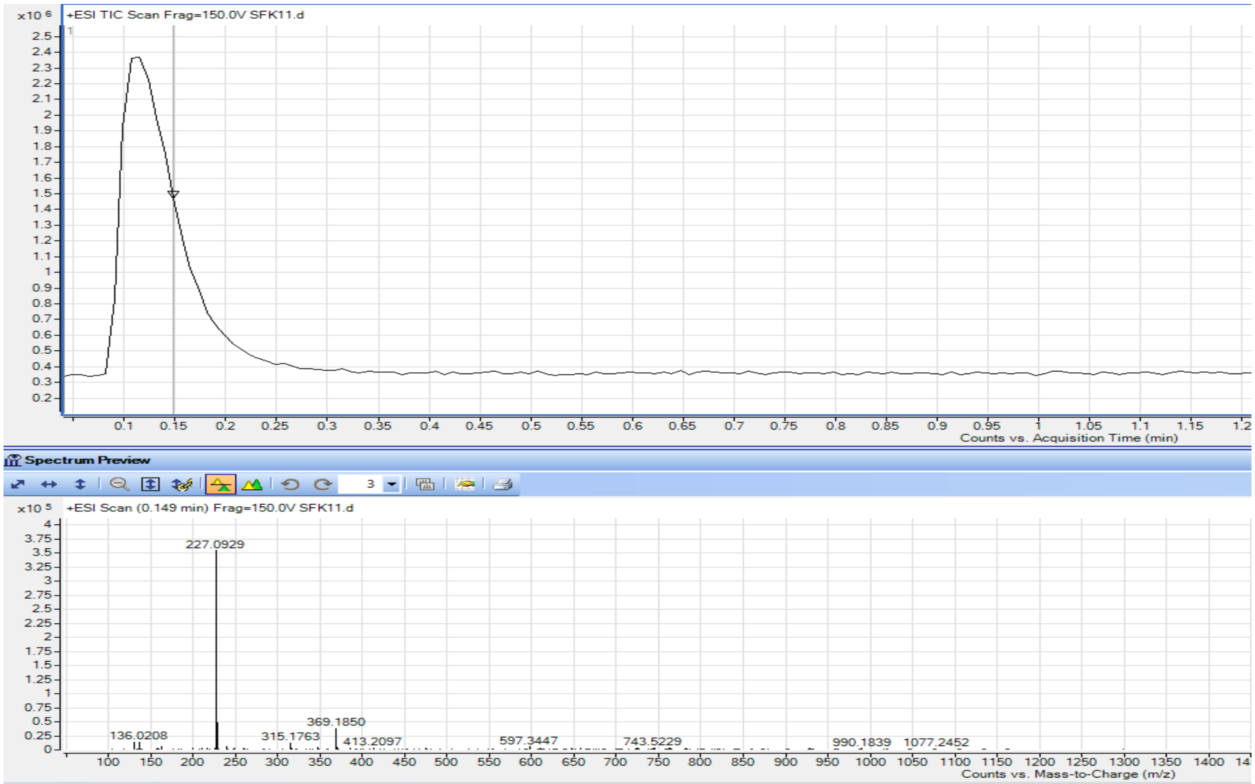

Figure S27. Meridianin A (**5**) HRSEIMS(+)

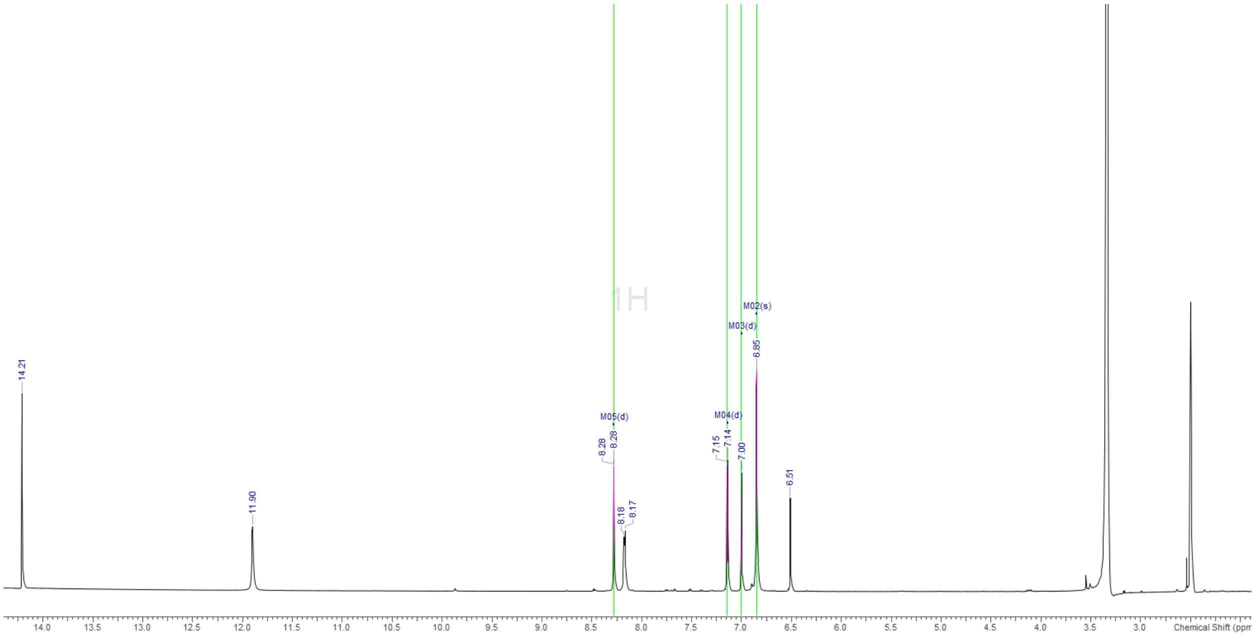

Figure S28. Meridianin B (**6**) ^{1}H NMR spectrum (400 MHz, DMSO-d_6)

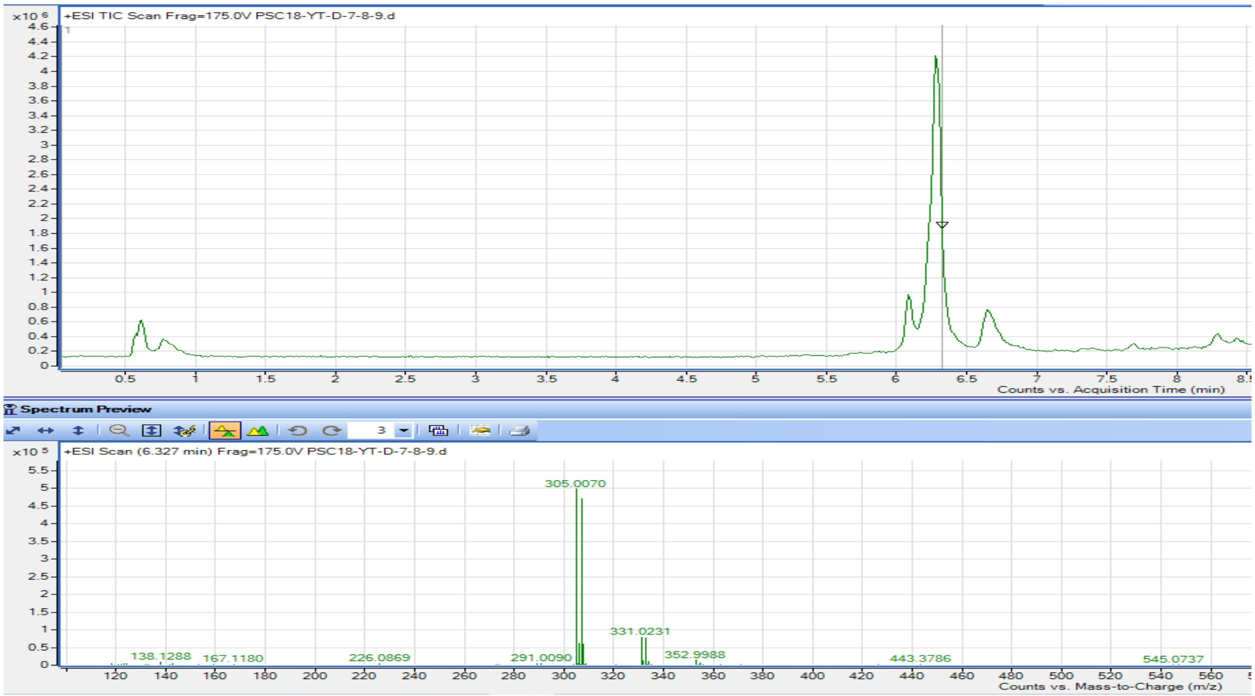

Figure S29. Meridianin B (**6**) HRESIMS

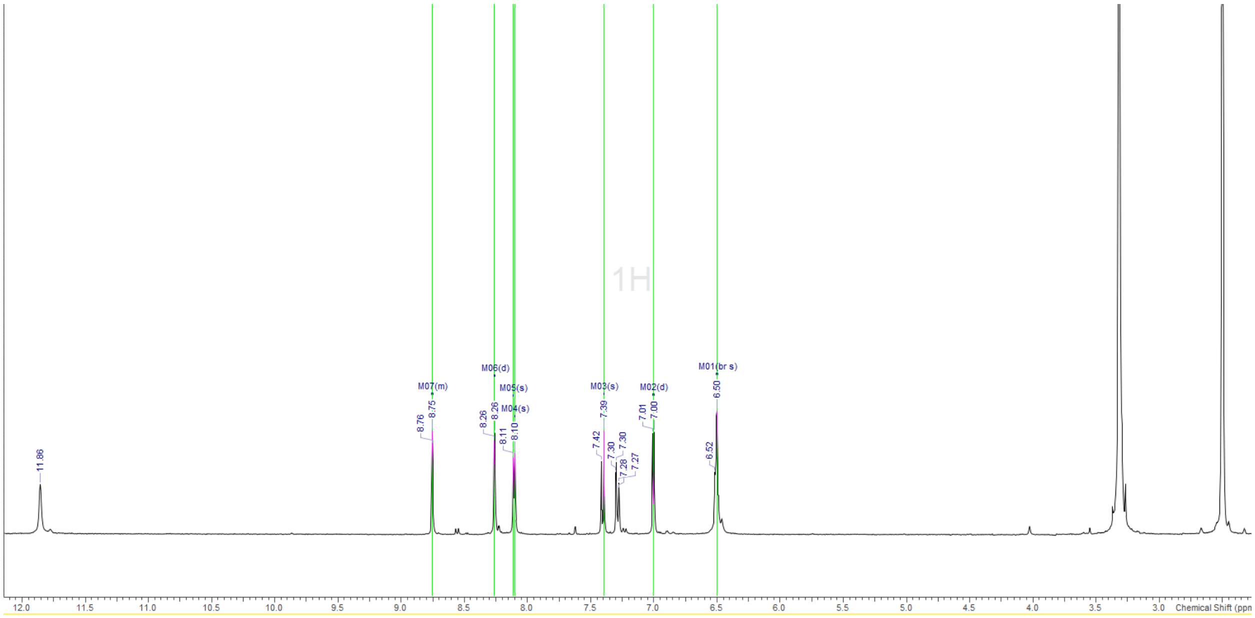

Figure S30. Meridianin C (**7**) ^{1}H NMR spectrum (400 MHz, DMSO-d_6)

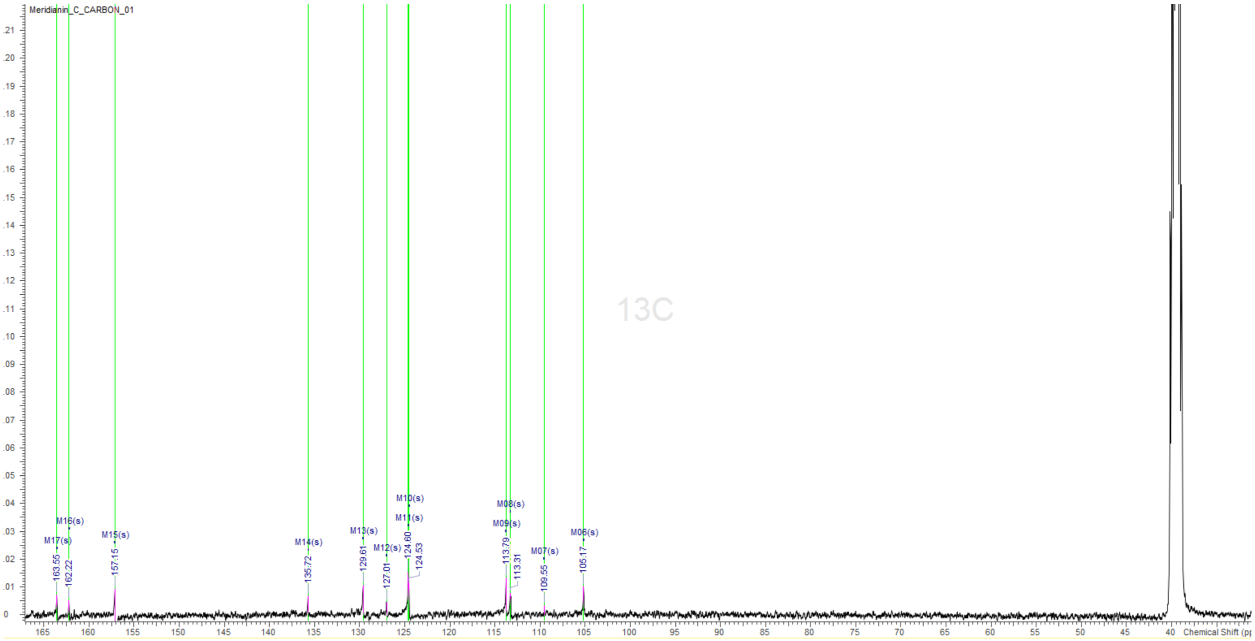

Figure S31. Meridianin C (**7**) ^{13}C NMR spectrum (100 MHz, DMSO-d_6)

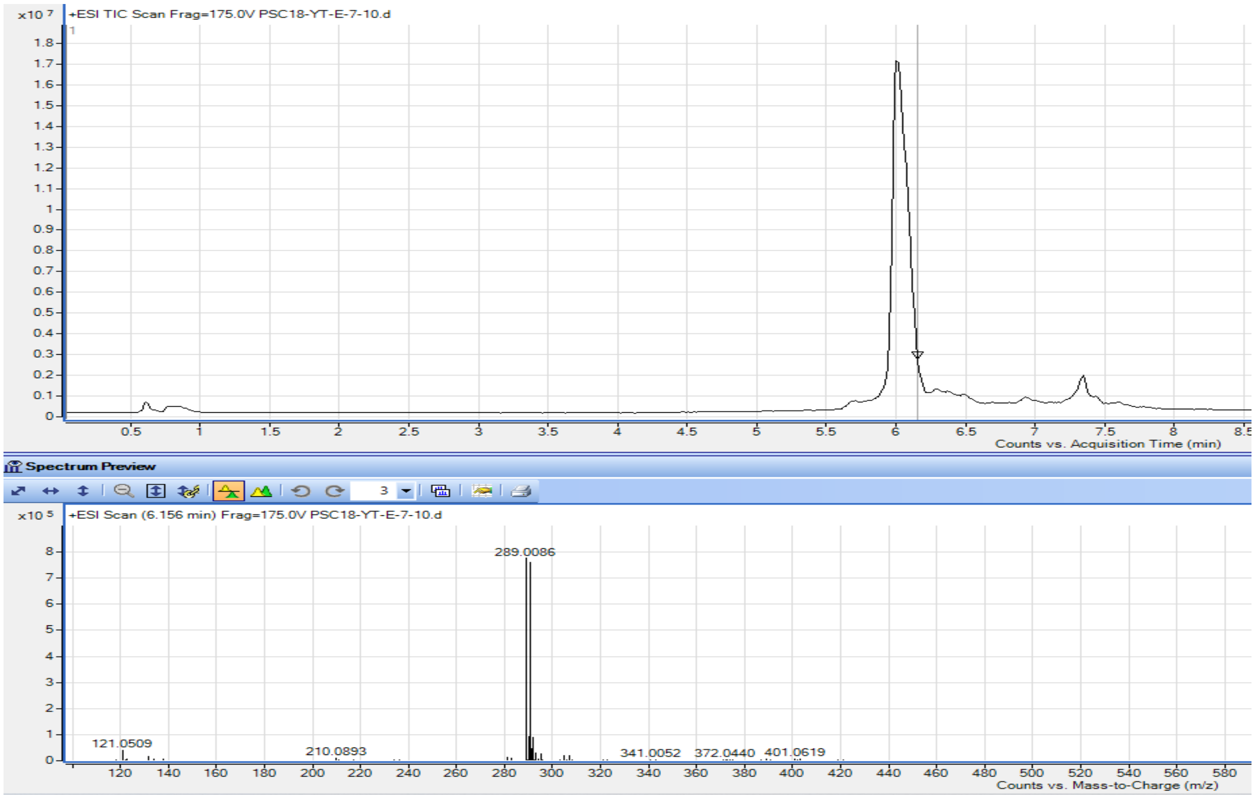

Figure S32. Meridianin C (7) HRESIMS(+)

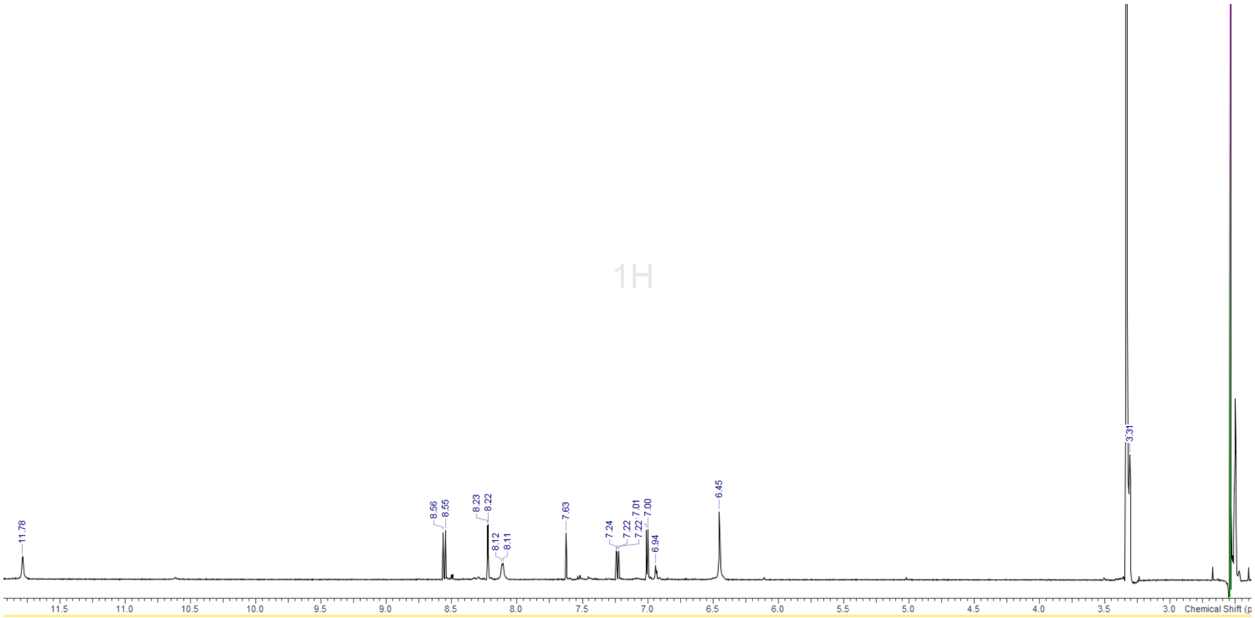

Figure S33. Meridianin D (**8**) ^{1}H NMR spectrum (400 MHz, DMSO-d_6)

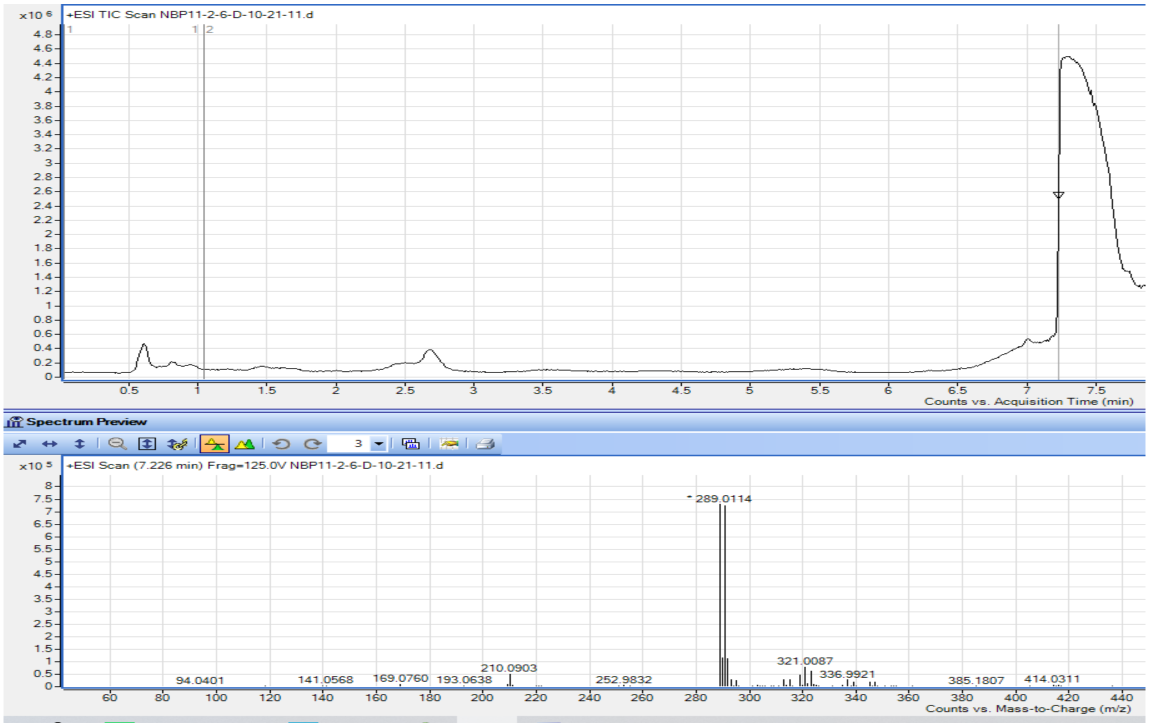

Figure S34. Meridianin D (**8**) HRESIMS(+)

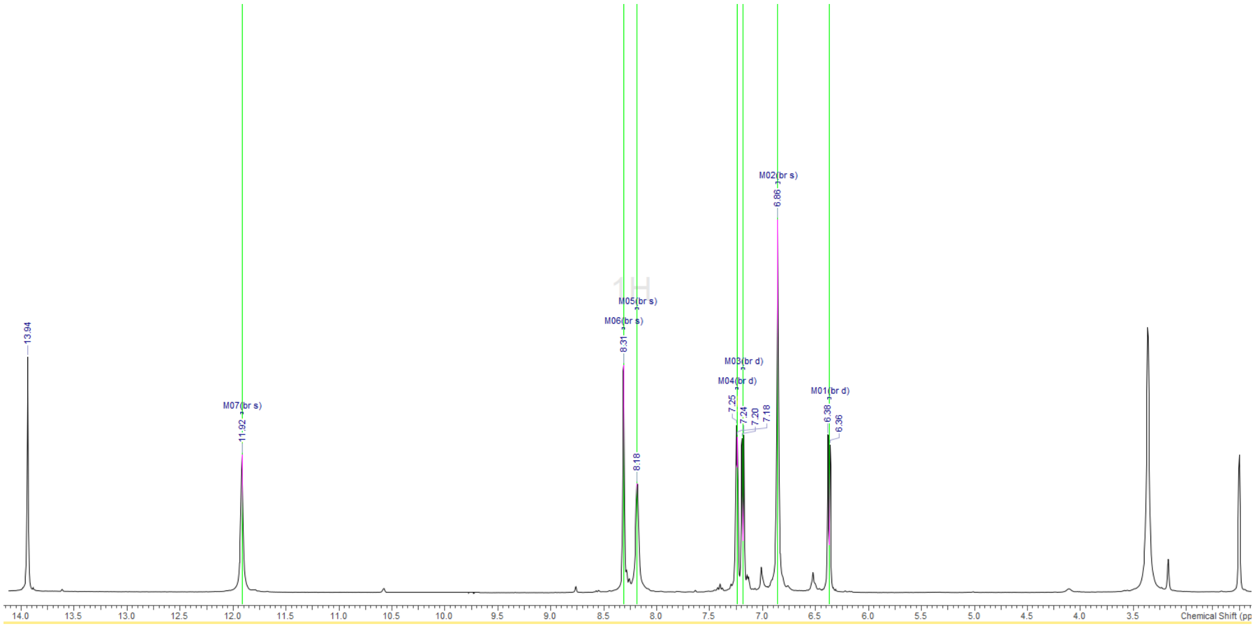

Figure S35. Meridianin E (**9**) ^{1}H NMR spectrum (400 MHz, DMSO-d_6)

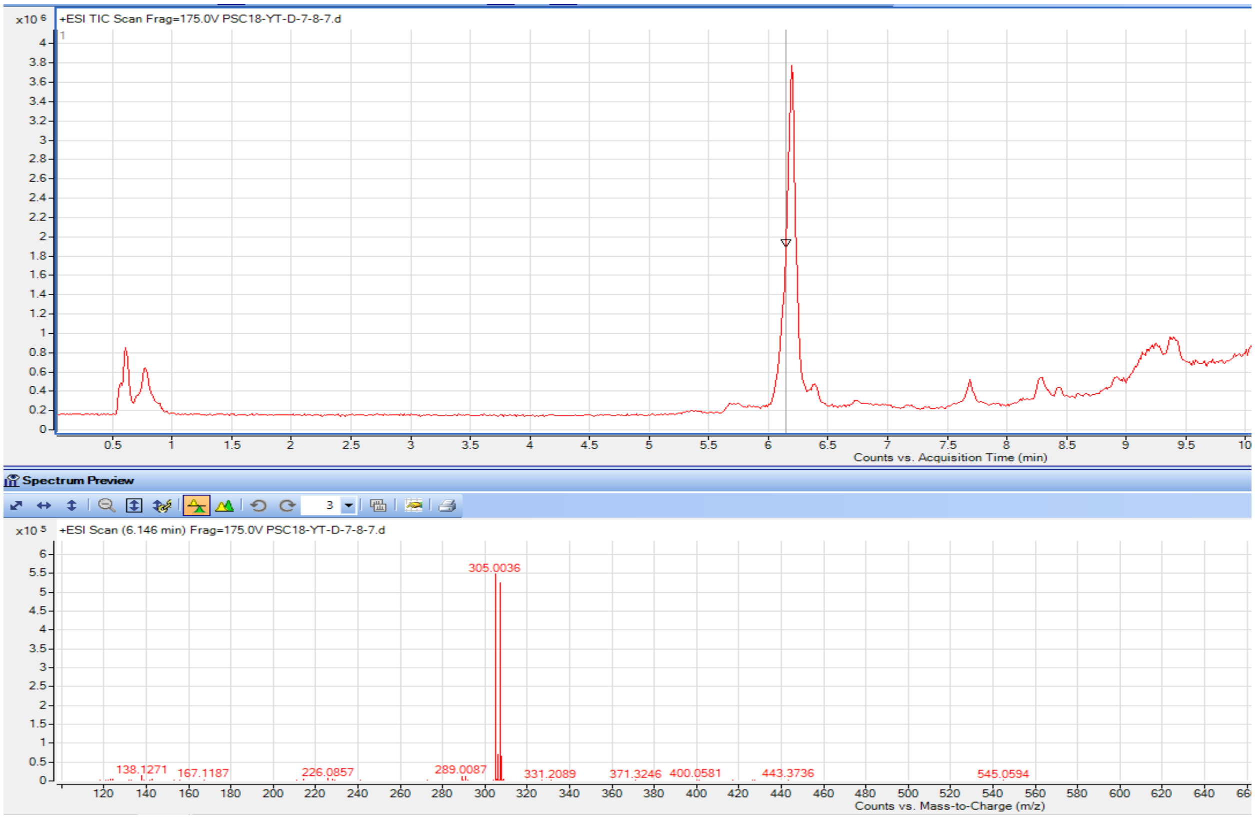

Figure S36. Meridianin E (**9**) HRESIMS(+)

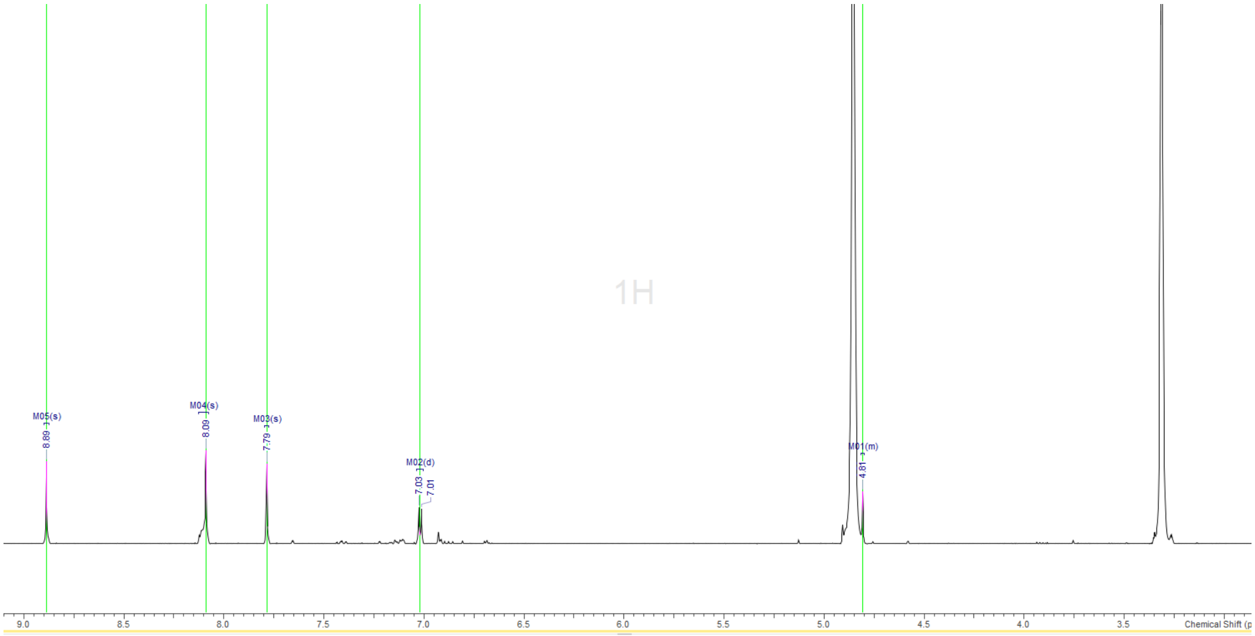

Figure S37. Meridianin F (**10**) ^{1}H NMR spectrum (400 MHz, CD_3OD)

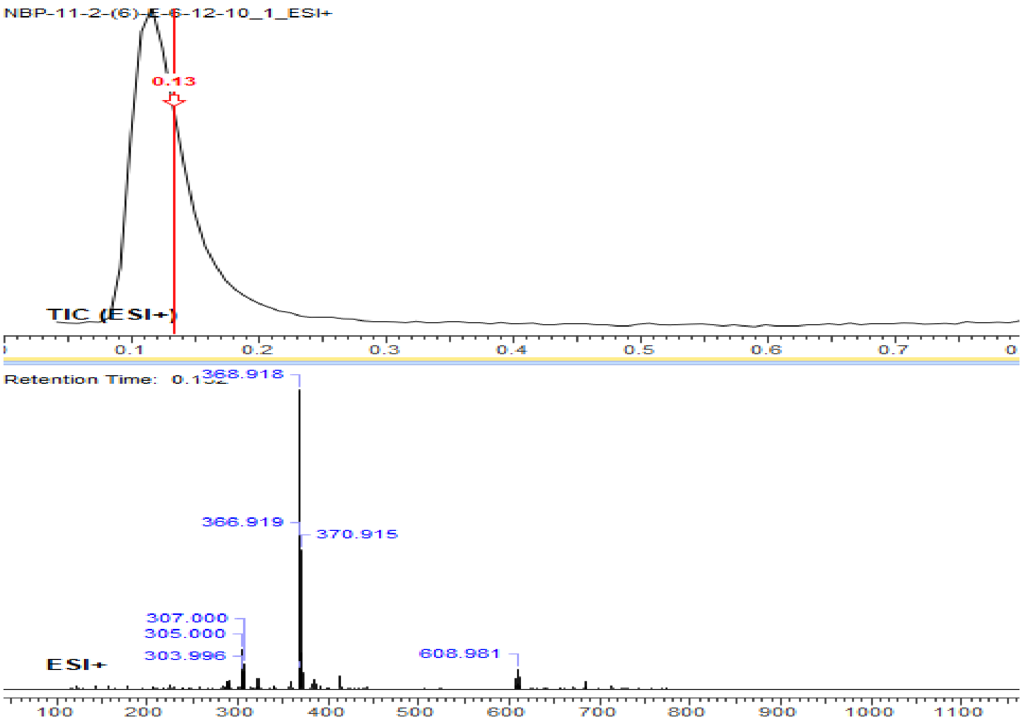

Figure S38. Meridianin F (**10**) HRESIMS(+)

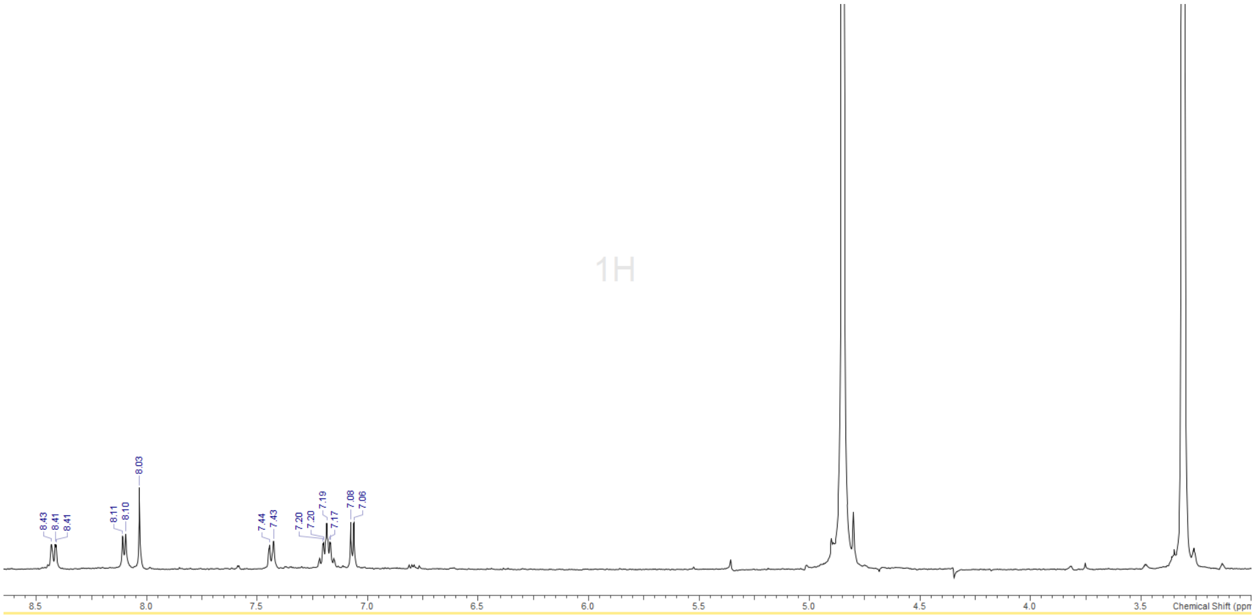

Figure S39. Meridianin G (**11**) ^{1}H NMR spectrum (400 MHz, CD_3OD)

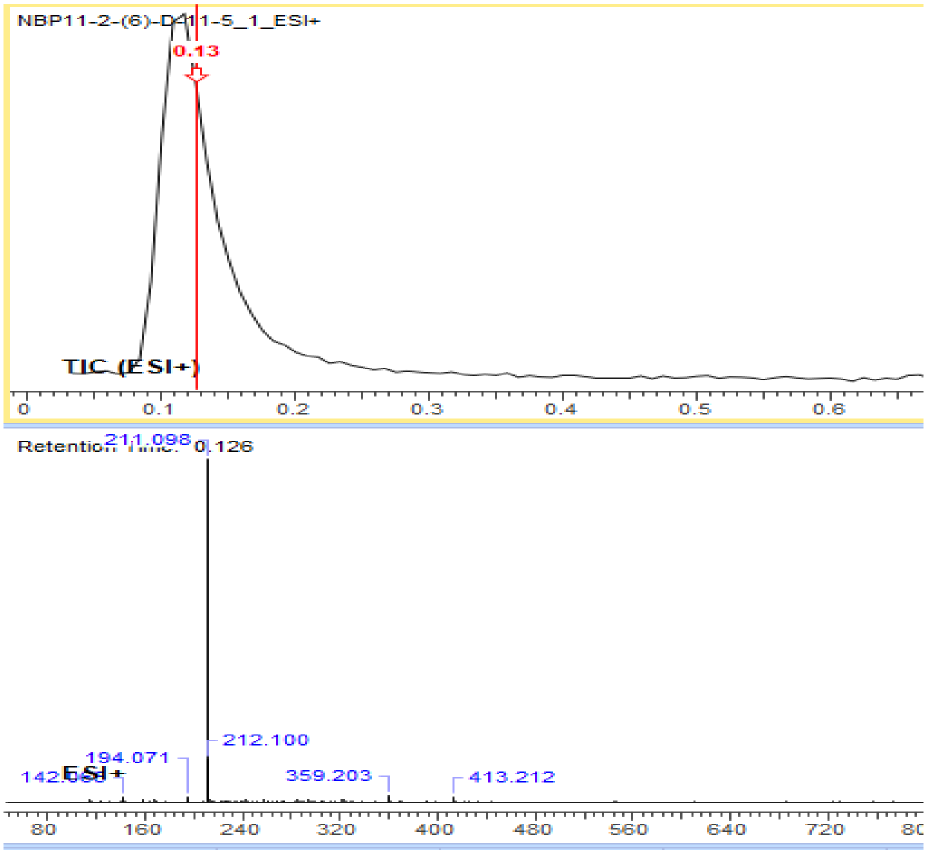

Figure S40. Meridianin G (**11**) HRESIMS(+)

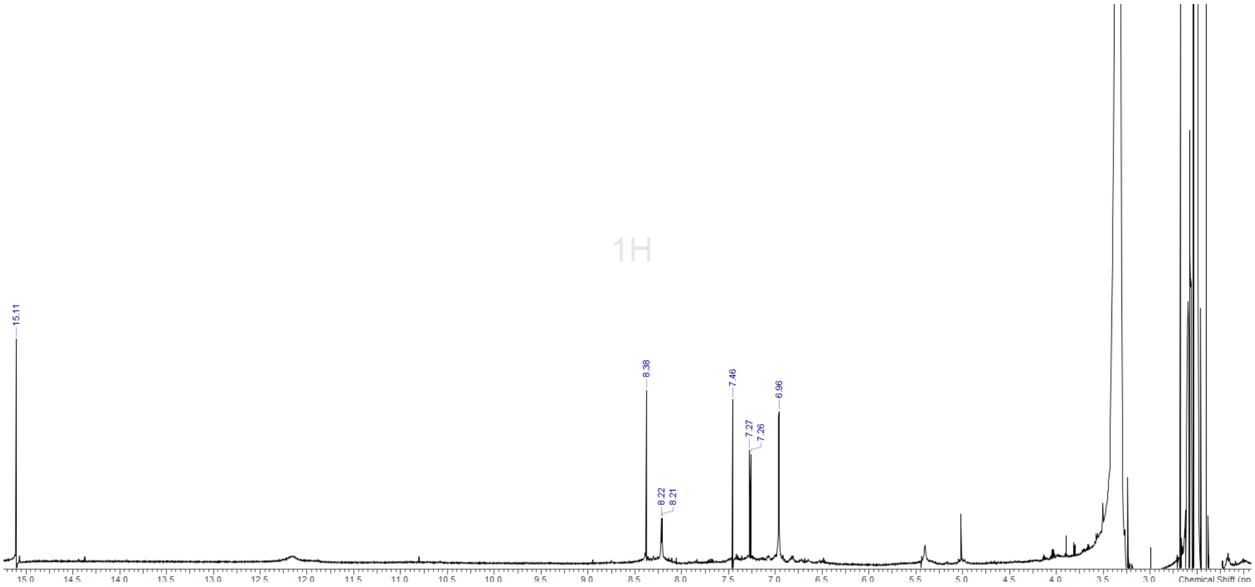

Figure S41. Meridianin H (**12**) ^{1}H NMR spectrum (500 MHz, DMSO-d_6)

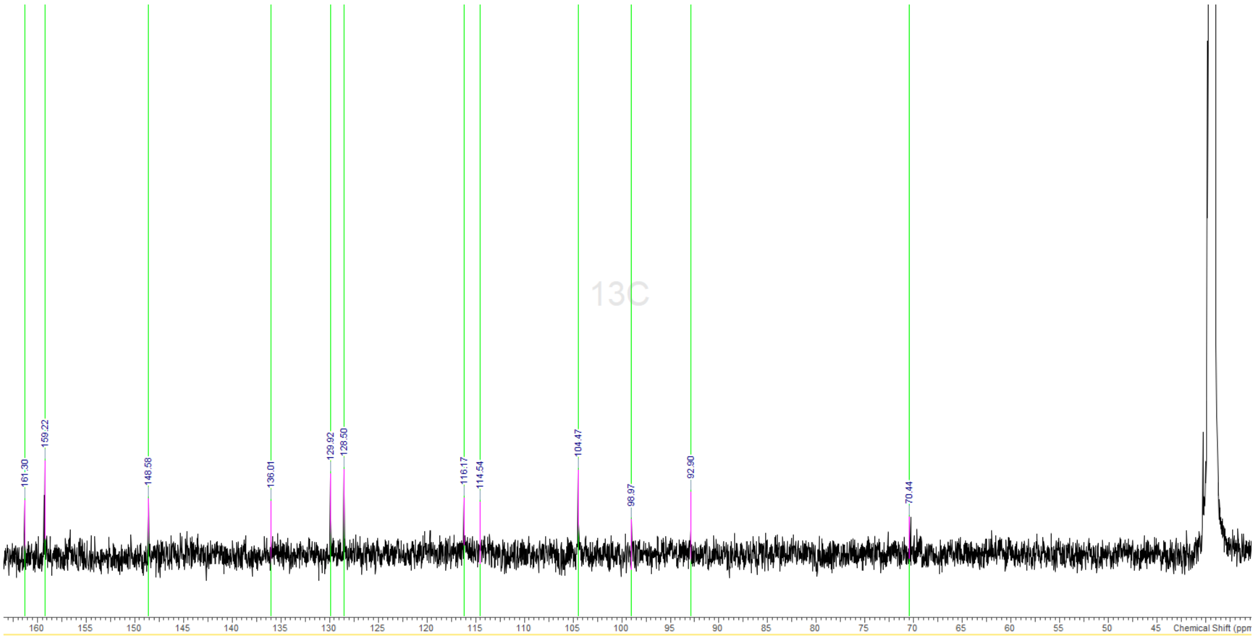

Figure S42. Meridianin H (**12**) ^{13}C NMR spectrum (125 MHz, DMSO-d_6)

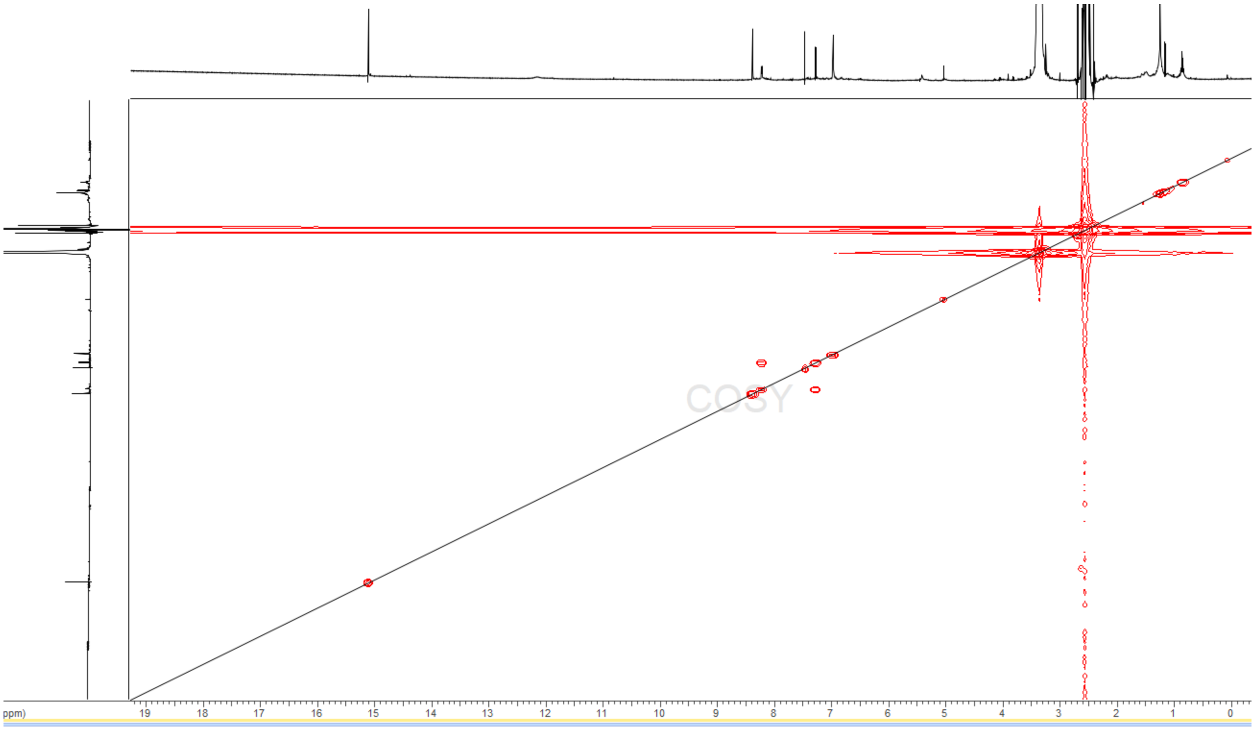

Figure S43. Meridianin H (**12**) COSY NMR spectrum (500 MHz, DMSO-d_6)

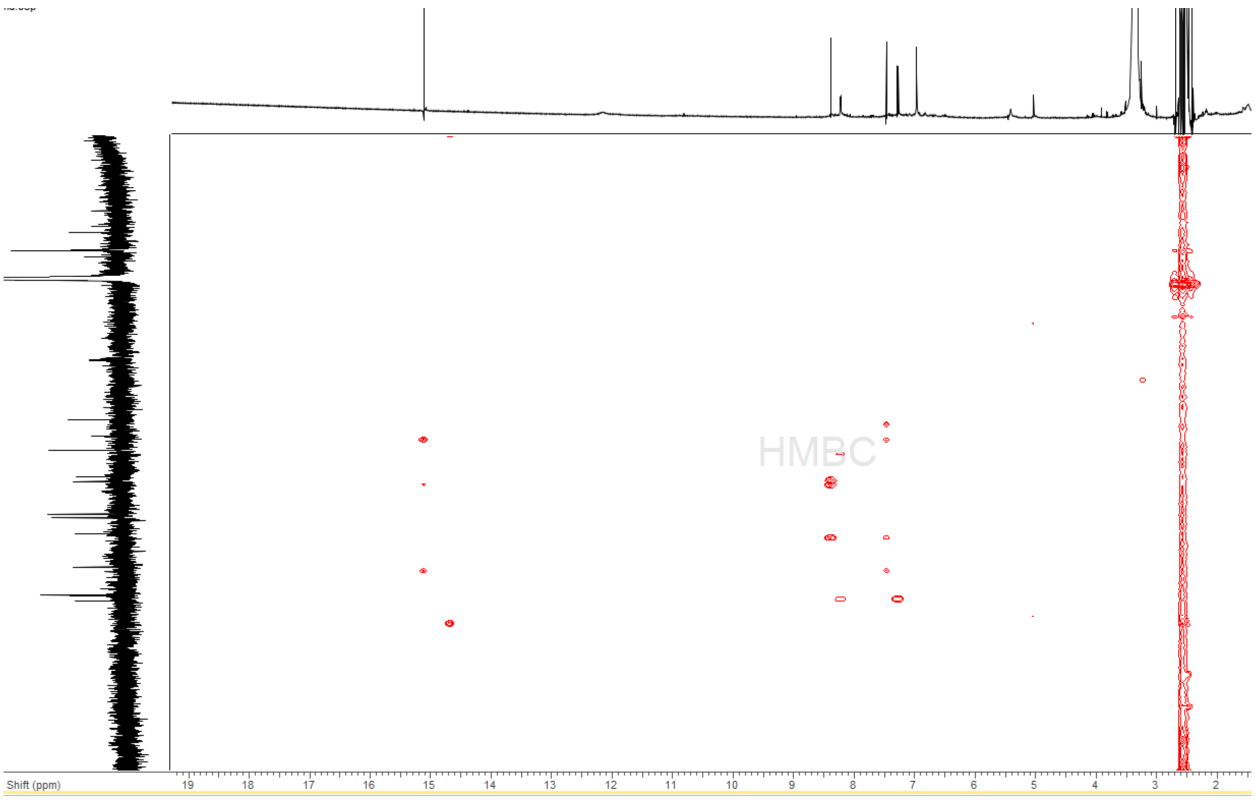

Figure S44. Meridianin H (**12**) HMBC NMR spectrum (500 MHz, DMSO-d_6)

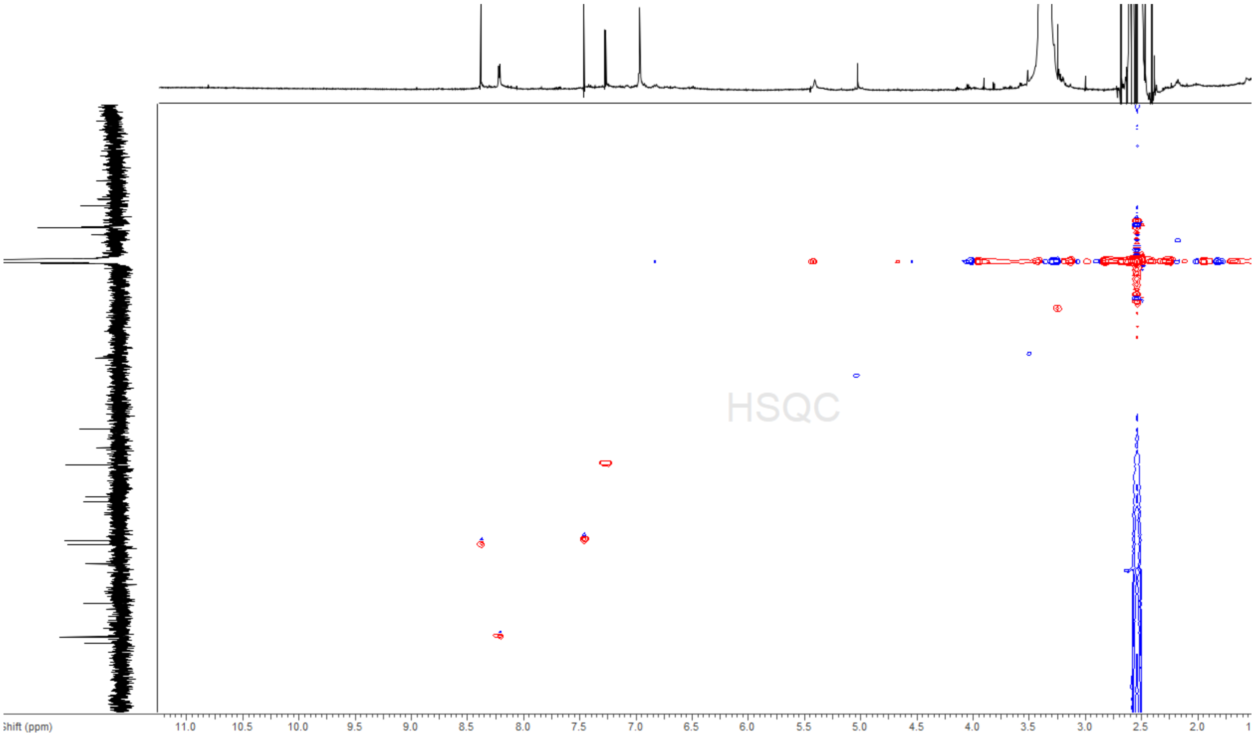

Figure S45. Meridianin H (**12**) HSQC NMR spectrum (500 MHz, DMSO-d_6)

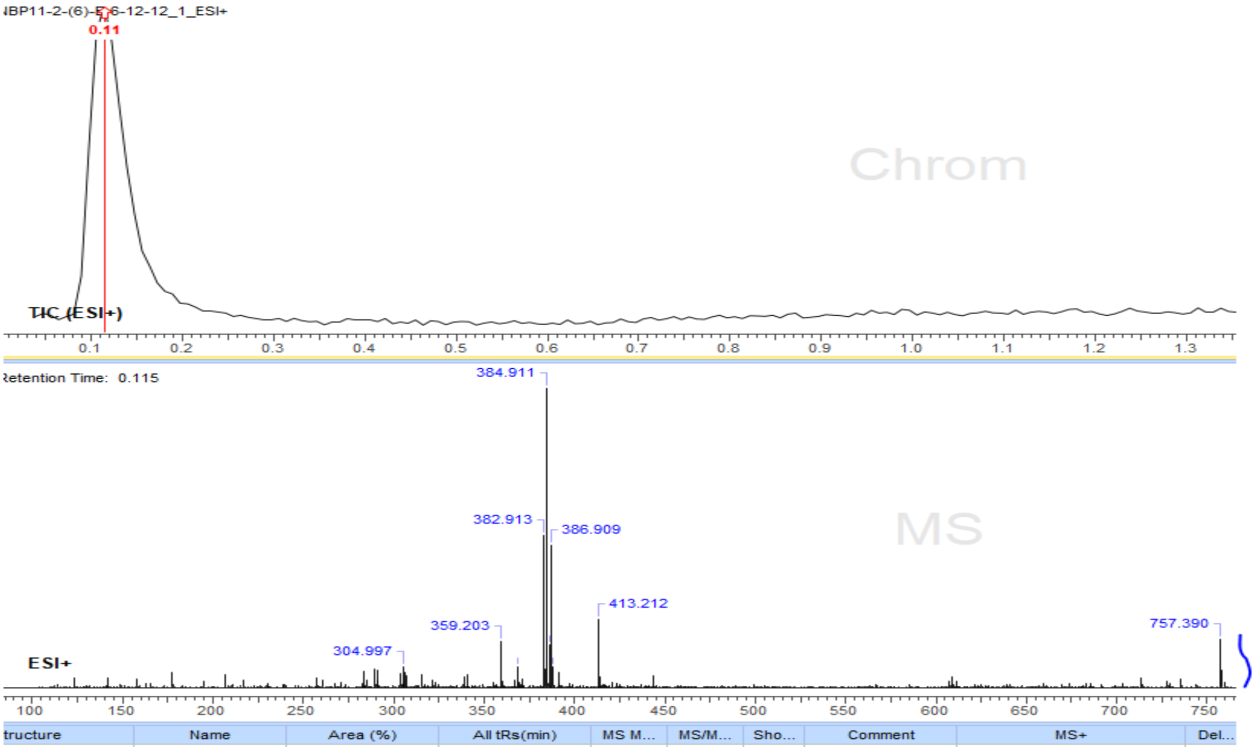

Figure S46. Meridianin H (**12**) HRESIMS(+)

Table S1. Crystal data and structure refinement for australindolone B (2).	
Identification code	NBP13_9G_8
Empirical formula	$C_{14}H_{17}BrN_4O_4S$
Moiety formula	$C_{12}H_9BrN_4O_2$, $(CH_3)_2SO$, H_2O
Formula weight	417.29
Temperature/K	100
Crystal system	orthorhombic
Space group	Pbcn
a/Å	27.1828(8)
b/Å	8.5340(3)
c/Å	14.9595(5)
α/°	90
β/°	90
γ/°	90
Volume/Å^3	3470.3(2)
Z	8
ρ_{calc}g/cm^3	1.597
μ/mm^{-1}	4.599
F(000)	1696.0
Radiation	CuKα (λ = 1.54178)
2Θ range for data collection/°	6.504 to 138.618
Index ranges	-32 ≤ h ≤ 32, -10 ≤ k ≤ 10, -17 ≤ l ≤ 17
Reflections collected	40465
Independent reflections	3224 [R_{int} = 0.0554, R_{sigma} = 0.0248]
Data/restraints/parameters	3224/552/349
Goodness-of-fit on F^2	1.171

Final R indexes [I>=2σ (I)]	R_1 = 0.0724, wR_2 = 0.1603
Final R indexes [all data]	R_1 = 0.0787, wR_2 = 0.1631
Largest diff. peak/hole / e $Å^{-3}$	0.52/-0.71
